# Pan-cancer analysis of immune checkpoint receptors and ligands in various cells in the tumor immune microenvironment

**DOI:** 10.18632/aging.206053

**Published:** 2024-08-07

**Authors:** Jiahuan Jiang, Yazhang Xu, Di Chen, Jiaxin Li, Xiaoling Zhu, Jun Pan, Leyi Zhang, Pu Cheng, Jian Huang

**Affiliations:** 1 Key Laboratory of Tumor Microenvironment and Immune Therapy of Zhejiang Province, Second Affiliated Hospital, Zhejiang University School of Medicine, Zhejiang University, Hangzhou 310009, China; 2 Department of Thyroid Surgery, The Second Affiliated Hospital, Zhejiang University School of Medicine, Zhejiang University, Hangzhou 310009, China; 3 Cancer Center, Zhejiang University, Hangzhou 310009, China; 4 Department of Gynecology, The Second Affiliated Hospital, Zhejiang University School of Medicine, Zhejiang University, Hangzhou 310009, China; 5 Department of Breast Surgery, The Second Affiliated Hospital, Zhejiang University School of Medicine, Zhejiang University, Hangzhou 310009, China; 6 Department of Neurology, The Fourth Affiliated Hospital, Zhejiang University School of Medicine, Zhejiang University, Yiwu 322000, China; 7 Department of Colorectal Surgery, The Fourth Affiliated Hospital, Zhejiang University School of Medicine, Zhejiang University, Yiwu 322000, China; 8 Liangzhu Laboratory, Zhejiang University Medical Center, Hangzhou 310009, China

**Keywords:** pan-cancer analysis, tumor immune microenvironment, ICRs, ICLs

## Abstract

Drugs that target immune checkpoint have become the most popular weapon in cancer immunotherapy, yet only have practical benefits for a small percentage of patients. Tumor cells constantly interact with their microenvironment, which is made up of a variety of immune cells as well as endothelial cells and fibroblasts. Immune checkpoint expression and blocked signaling of immune cells in the tumor microenvironment (TME) are key to tumor progression. In this study, we perform deliberation convolution on the TCGA database for human lung, breast, and colorectal cancer to infer crosstalk between immune checkpoint receptors (ICRs) and ligands (ICLs) in TME of pan-carcinogenic solid tumor types, validated by flow cytometry. Analysis of immune checkpoints showed that there was little variation between different tumor types. It showed that CD160, LAG3, TIGIT were found to be highly expressed in CD8+ T cells instead of CD4+ T cells, PD-L1, PD-L2, CD86, LGALS9, TNFRSF14, LILRB4 and other ligands were highly expressed on macrophages, FVR, NECTIN2, FGL1 were highly expressed on Epithelial cells, CD200 was highly expressed in Endothelial cells, and CD80 was highly expressed in CD8 High expression on T cells. Overall, our study provides a new resource for the expression of immune checkpoints in TME on various types of cells. Significance: This study provides immune checkpoint expression of immune cells of multiple cancer types to infer immune mechanisms in the tumor microenvironment and provide ideas for the development of new immune checkpoint-blocking drugs.

## INTRODUCTION

Tumor microenvironment (TME) has been the focus of tumor prognosis research in recent years, in which immune cells and stromal cells are two important components of TME. Recent studies have shown that immune cell infiltration in TME leads to host-tumor interactions. Immune cells play an integral role in cancer progression and treatment response and are important prognostic factors for cancer [1–3]. Immunotherapy has shown strong anti-tumor activity in the treatment of breast, liver, ovarian and other cancers, and was therefore named the most important scientific breakthrough of the year by Science magazine in 2013 [4, 5]. The U.S. Food and Drug Administration (FDA) has approved several tumor immunotherapy drugs for clinical use.

**Figure 1:**
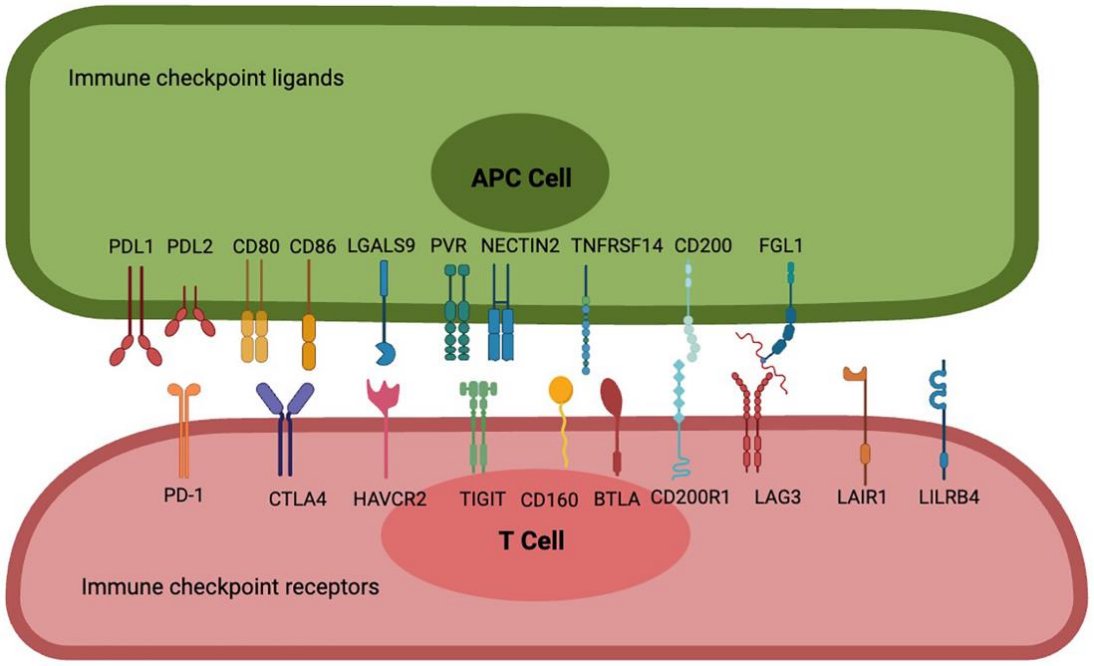
**Interactions between frontier immune checkpoint receptors and corresponding ligands.** PD-1 is a potent T cell inhibitory receptor that binds to PDL1 or PDL2 to inhibit T cell activation, differentiation, and proliferation, resulting in an immunotolerant state; CTLA-4 has a significant strong affinity for CD80 and CD86, leading to blockade of CD80 and CD86 co-stimulation and inhibition of sustained T cell activation; Cell 1 (Th1) apoptosis, resulting in reduced autoimmune and anti-tumor immune responses. T cell immune receptors with TIGIT are thought to activate inhibitory receptors in T cells, NK cells, and regulatory T cells (Tregs), including NECTIN2 and high-affinity homologous receptor PVRs. CD160 binds broadly but weakly to MHC class I molecules, binds strongly to HVEM to attenuate the activity of specific subsets of CD4 T lymphocytes or enhance the activity of CD8 T cells, and also controls cytokine production in NK cells, while BTLA has two immunoreceptor tyrosine-based inhibitory motifs, and binding to HVEM is involved in providing an overall inhibitory immune response. CD200 is a type 1 cell membrane glycoprotein of the immunoglobulin supergene family, and its interaction with its receptor CD200R leads to the attenuation of multiple immune responses, FGL1, as a novel checkpoint ligand that surpasses the LAG3 classical ligand MHC II, is a proliferation and metabolism-related protein secreted by the liver, and their binding transmits negative signals to activated T cells to prevent immune-mediated tissue damage, and LAIR-1, a novel immunosuppressive transmembrane protein. Interactions with several of its ligands (e.g., extracellular matrix collagen, C1q complement components, surfactant protein D, and adiponectin) induce phosphorylation of ITIM and inhibit immune cell activation or differentiation, while LILRB4 binds to ligands such as ALCAM, ApoE, fibronectin, Galectin-8 and others to inhibit antigen-presenting cell activation, leading to immune tolerance.

With the application of immune checkpoint blockers (ICBs), scientists have come to realize that the immune activation generated by targeting programmed cell death 1 (PD-1) combined with PDL1and PDL2 [6], or cytotoxic T-lymphocyte associated protein 4 (CTLA-4) combined with CD80 and CD86 [7], is not enough to control tumor progression. Based on the conclusions of current preclinical and clinical studies, we can preliminarily see that the most promising targeted immune checkpoint receptors and ligands include, but are not limited to: lymphocyte-activation gene-3 (LAG-3) combined with fibrinogen like 1 (FGL1) [8, 9], T-cell immunoglobulin and ITIM domain (TIGIT) combined with poliovirus receptors (PVR) and nectin cell adhesion molecule 2 (NECTIN2) [10–12], Hepatitis A virus cellular receptor 2 (HAVCR2) combined with lectin galactoside-binding soluble 9 (LGALS9) [12–15], CD200 receptor 1 (CD200R1) combined with CD200 [16–20], CD160 combined with TNF receptor superfamily member 14 (TNFRSF14) [21–24], B and T lymphocyte associated (BTLA) combined with TNFRSF14 [22, 23, 25, 26], Leukocyte associated immunoglobulin like receptor 1 (LAIR1) [27–30] mainly combined with collagen, and leukocyte immunoglobulin like receptor B4 (LILRB4) with a variety of ligands [31–33]. We will cover these immune checkpoint molecules and highlight their expression at different tumor, at different cellular levels. The mechanism of interaction between these immune checkpoints is clearly and schematically described (Figure 1).

We know roughly from previous research that PD-1 is expressed on the surface of various immune cells, including activated CD4+ T cells, CD8+ T cells, B cells, natural killer T cells, monocytes, and dendritic cells DCs) while PD-L1 and PD-L2 are mainly expressed in tumor cells [34]. CTLA-4 is thought to be expressed only on T cells [35], CD80 and CD86 mainly localizing to cell membranes and rarely expressing in tumor interstitium, are expressed in activated B lymphocytes, activated T lymphocytes, macrophages, peripheral blood mononuclear cells, and DCs [36]. LAG3 is expressed in CD4 and CD8+ T cells, as well as T regs, and is required for optimal T cell regulation and homeostasis [37], and FGL1 was found to be highly expressed in human cancer cells [8]. TIGIT is an inhibitory receptor expressed on lymphocytes including T cells and B cells [38], while its ligand PVR is mainly expressed on T cells and natural killer cells [39], at the same time another ligand, NECTIN2, is highly expressed in epithelial tumors but low in non-epithelial tumors [40]. HAVCR2 is a novel immune checkpoint protein recently discovered in different immune-associated cells, including T cells, Tregs, DCs, and macrophages [41–43], and LGALS9 is mainly expressed in immune cells, especially myeloid cells, including dendritic cells and monocytes [44]. In humans and mice, CD200R1 is expressed predominantly on myeloid cells monocytes, granulocytes, macrophages, and DCs), but also on natural killer cells NK) and T lymphocyte and B lymphocyte subsets [43, 45], while CD200 is predominantly expressed on B cells, DCs, and activated T cells, as well as vascular endothelial cells and many non-hematopoietic cell types, including cells within the central nervous system and retina neurons) [46]. CD160 is highly expressed in CD56dimCD16 NK cells, NKT cells, γδ T cells, CD8+CD28− T cells, a small subset of CD4+ cells, and intestinal intraepithelial T cells, and is aberrantly expressed in B-cell chronic lymphocytic leukemia, but not in normal B lymphocytes [47, 48], while TNFRSF14 is widely expressed on APCs, endothelial cells, and lymphocytes, with the highest expression on naïve T cells [49]. For BTLA, it is expressed on T, B, and NK cells, as well as DC and endothelial cells, and is especially highly expressed on impotent T cells [50]. LAIR-1 is expressed on most peripheral monocytes, including NK cells, T cells, B cells, monocytes, and dendritic cells [51]. LILRB4 is mainly expressed in the myeloid lineage, such as monocytes, macrophages, neutrophils, and dendritic cells, and to a lesser extent on B cells, NK cells, and T cells [52].

However, the expression patterns of these ICRs and ICLs and their prognostic potential have not been studied from a pan-cancer perspective. And most current studies are based on a single cell subset or the interaction between two cells when studying the relationship between immune checkpoints and ligands, without macroscopic analysis of the expression of various cells in the immune microenvironment. The purpose of this study was to study the expression of multiple immune checkpoints and ligands in various immune cells and stromal cells in the tumor immune microenvironment, and to evaluate the role of different immune checkpoints in tumor immunotherapy. First, we searched the TCGA databases of breast cancer, colorectal cancer, and lung cancer, and performed bioinformatics analysis of 20 immune checkpoints and ligand expressions of 13 types of cells, and then used clinical fresh samples of breast cancer, colorectal cancer, and lung cancer for flow cytometry analysis. Finally, we uncover the effects of different immune checkpoints on different cells in the tumor immune microenvironment and provide potential therapeutic strategies for tumor immunotherapy. In conclusion, our study elucidates the differences in the expression of different immune checkpoints and ligands on different cells in different tumors and provides new possibilities for identifying better targets for tumor immunotherapy.

## RESULTS

### Distribution of immune cells and stromal cells in tumors

**Figure 2:**
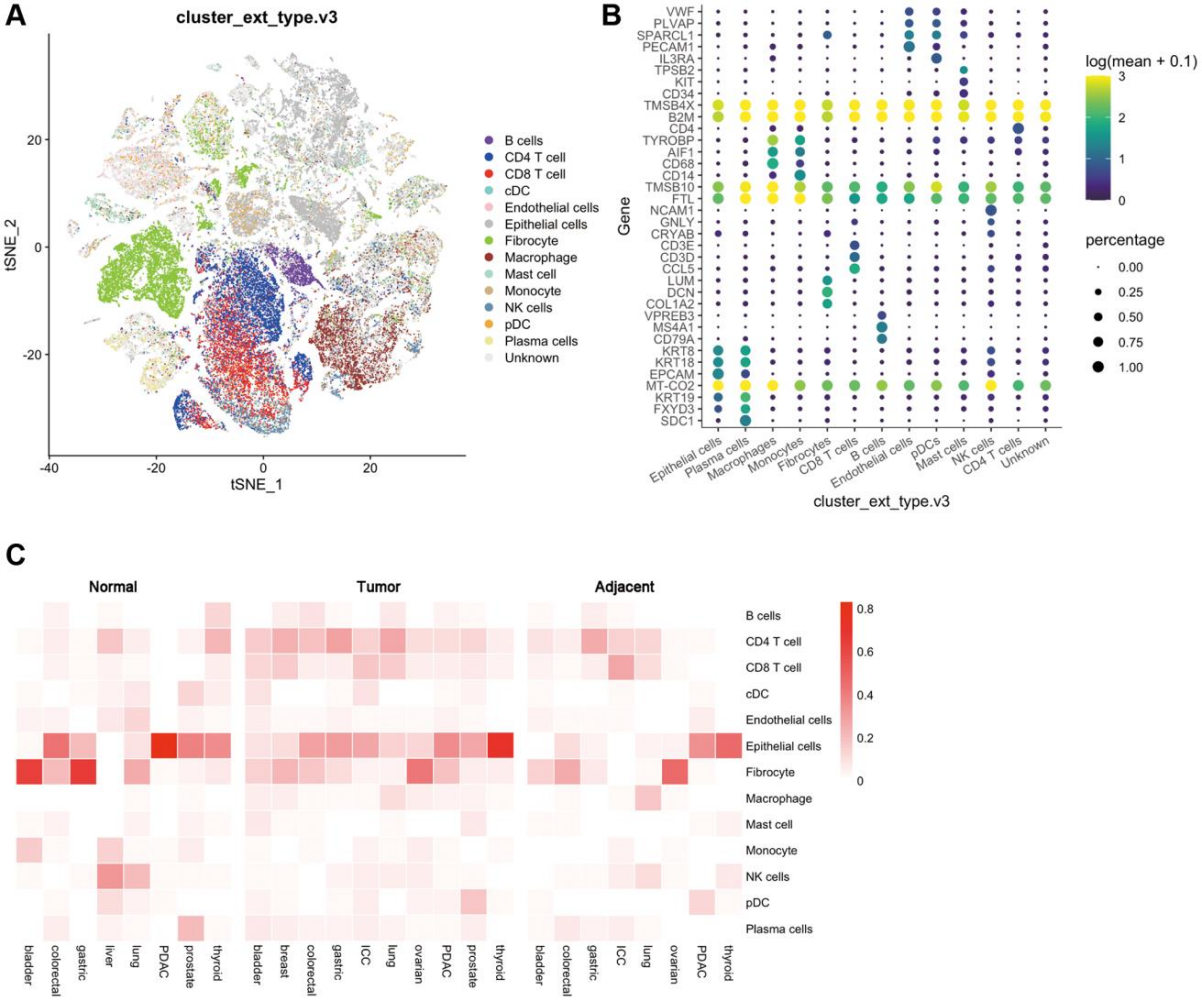
**Distribution of various cells and immune checkpoints in the tumor immune microenvironment.** (**A**) t-SNE plots of tumor-infiltrating T cells and stromal cells, overlaid with color-coded clusters; (**B**) The expression of immune genes in different types of immune cells. The size of the dots represents the proportion of cells with positive gene expression, and the color represents the level of gene expression; (**C**) Heat map of the distribution ratio of cells in the immune microenvironment in normal tissues, adjacent tissues, and tumor tissues of a variety of tumors.

First, the GEO database was used to analyze the expression of cell subsets in multiple cancer species. The results showed that 13 types of cells, including B cells, Plasma cells, CD4+ T cells, CD8+ T cells, classical dendritic cells cDCs), plasmacytoid dendritic cells pDCs), endothelial cells, epithelial cells, fibrocyte, macrophage, mast cells, monocyte, NK cells, could be detected in all the multi-cancer species evaluated Figure 2A, 2B).

Then, the GEO database was used to evaluate the expression differences of these 13 cells in various types of tumors. Normal tissues and adjacent tissues were used as controls for differential expression analysis. We found that in bladder cancer, the expression of CD4 T cells, CD8 T cells, cDCs, epithelial cells, macrophages, mast cells, and plasma cells showed an up-regulated trend, while the expression of B cells, fibroblasts, and monocytes showed a down-regulated trend. Breast cancer lacks a control between normal tissues and adjacent tissues, and its immune microenvironment is dominated by CD4 T cells, CD 8T cells, fibroblasts, epithelial cells, macrophages and B cells. In colorectal cancer, B cells, CD4 T cells, CD8 T cells are upregulated, and epithelial cells are downregulated. In gastric cancer, CD4 T cells are upregulated and fibroblasts and plasma cells are downregulated. And in ICC, cDCs, epithelial cells, and pDCs are upregulated while B cells are downregulated. In lung cancer, B cells, CD4 T cells, and CD8 T cells are up-regulated, while cDC, endothelial cells, fibroblasts, macrophages, and NK cells are down-regulated. In ovarian cancer, CD4 T cells, CD8 T cells, and epithelial cells were upregulated, and there were no significant differences in other cell subsets. In PDAC, B cells, CD4 T cells, CD8 T cells, endothelial cells, and fibroblasts are upregulated, and epithelial cells are downregulated. In prostate cancer, B cells, CD4 T cells, CD8 T cells, mast cells, and pDCs are upregulated, and cDCs, endothelial cells, monocytes, and plasma cells are downregulated. Finally in thyroid cancer, epithelial cells are upregulated, and B cells, CD4 T cells, CD8 T cells, cDCs, and NK cells are downregulated (Figure 2C).

### Expression levels of immune checkpoint inhibitors in pan-cancer

**Figure 3:**
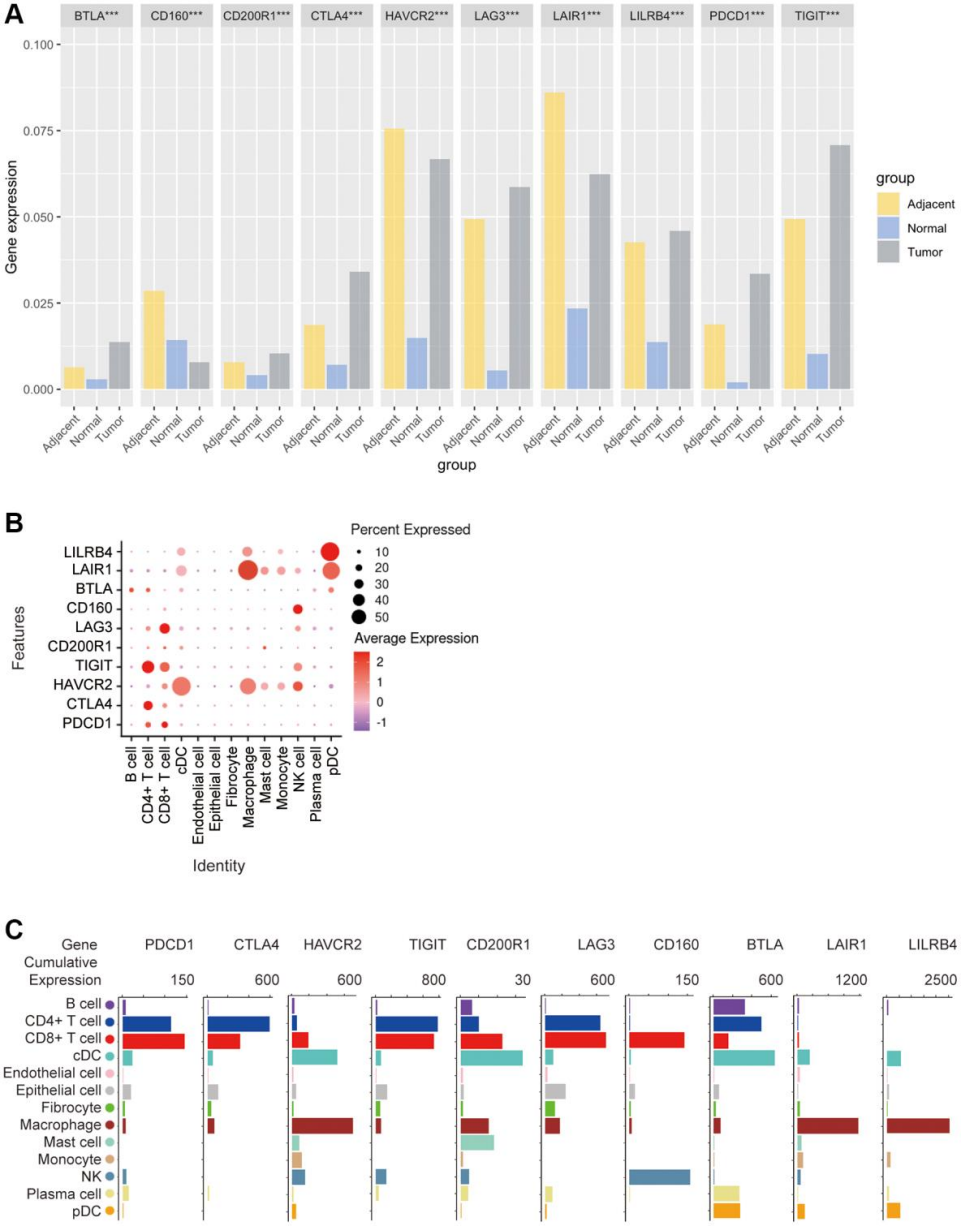
**Bioinformatics analysis of the distribution of 10 immune check site receptors on tissues and cells.** (**A**) Expression ratios of 10 immune checkpoints receptors in tumors, blood, spleen, lung, muscle, and bone marrow tissues; (**B**) Bioinformatics analysis of the expression of 10 immune checkpoint receptors in 13 cell subsets showed a heat map of the normalized markers for each cluster, with data representing the entire tumor; (**C**) Bioinformatics analysis of receptor expression at 10 immune checkpoints, histogram showing high and low expression of each receptor on different cell subsets, and data representing the entire tumor.

Then, the GEO database was used to analyze the expression of these 10 ICRs in tumor, adjacent and normal tissues. The results showed that the expression of TIGIT, PD1, LILRB4, LAG3, CTLA4, CD200R1 and BTLA was the highest in tumor tissues, the expression of LAIR1 and HAVCR2 in tumor tissues was lower than that of adjacent tissues but higher than that of normal tissues, and the expression of CD160 was the lowest in tumor tissues. A very special finding was that all kinds of receptors were least expressed in normal tissues, with the exception of CD160, which was expressed more in normal tissues than in tumor tissues (Kruskal-Wallis test, *p* < 0.001) (Figure 3A).

In order to explore the correlation between ICRs expression and specific types of cell infiltration in the tumor immune microenvironment, the relative abundance of 13 cell types was inferred by gene set variant analysis (GSVA) based on the ICRs expression profile on TISIDB. The results showed that in n TCGA tumors, PD-1 expression was closely related to CD4+ T cells and CD8+ T cells, CTLA4 expression was closely related to CD4+ T cells, HAVCR2 expression was closely related to cDCs and macrophages, TIGIT expression was closely related to CD4+ T cells and CD8+ T cells, CD200R1 expression was closely related to cDC, LAG3 expression was closely related to CD8+ T cells, and CD160 expression was closely related to NK cellsBTLA expression was closely related to B cells, CD4+ T cells and pDCs, LAIR1 expression was closely related to macrophages and pDCs, and LILRB4 expression was significantly related to macrophages and pDCs (Figure 3B).

In addition, we again analyzed the correlation between the expression of these 10 ICRs and the gene expression of 13 cell subsets using other methods. The results showed that the expression trends of PD-1, TIGIT and LAG3 were similar in n TCGA tumors, with the highest expression levels in CD4+ T cells and CD8+ T cells, a small amount in B cells, cDCs, epithelial cells, fibroblasts, macrophages, NK and plasma cells, and almost no expression in endothelial cells, mast cells, monocytes and pDCs, and the expression trend of CTLA4 was similar to that of PD-1, except that PD-1 was expressed in CD8+ T cells. The highest expression was on T cells, while CTLA4 was highest on CD4+ T cells, and CTLA4 was almost non-expressed on NK cells, HAVCR2 was highly expressed on cDCs and macrophages, and almost no expression on endothelial cells and plasma cells, in addition, CD200R1 was the highest expression on cDCs, followed by CD8+ T cells, macrophages and mast cells, and CD160 was expressed only in CD8+ BTLA was most expressed on cDCs, followed by CD4+ T cells, and moderately expressed on B cells, plasma cells, pDCs, and CD8+ T cells, and finally LAIR1 and LILRB4 had a similar expression trend, with only high expression on macrophages and a small amount on other cells (Figure 3C).

**Figure 4:**
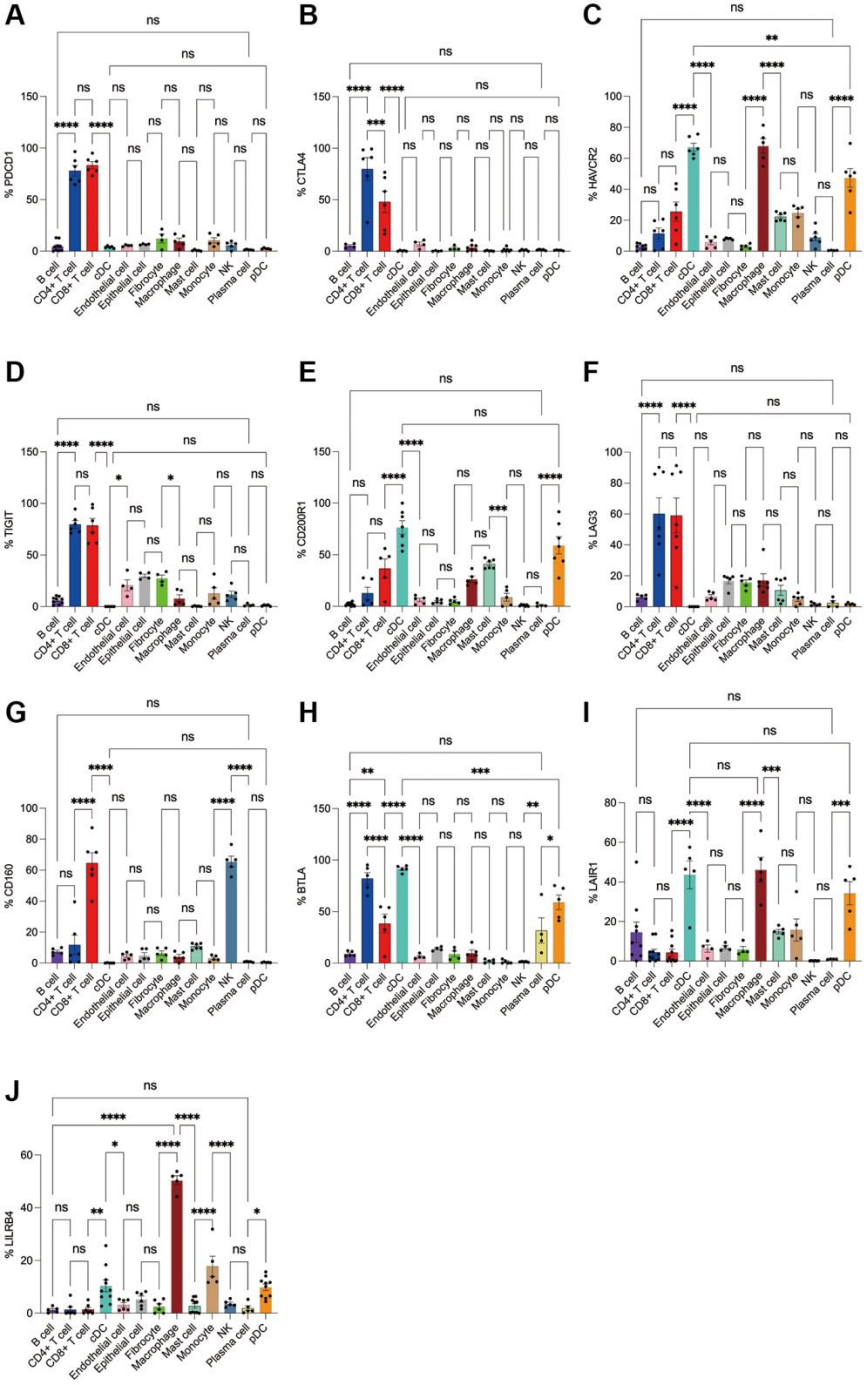
**Flow cytometry analysis of the differences in the expression proportions of 10 immune checkpoint receptors in 13 cell subsets in human breast cancer.** (**A**) Flow cytometry analysis of the expression ratio of PD-1 in 13 cell subsets; (**B**) Flow cytometry analysis of the expression ratio of CTLA4 in 13 cell subsets; (**C**) Flow cytometry analysis of the expression ratio of HAVCR2 in 13 cell subsets; (**D**) Flow cytometry analysis of the expression ratio of TIGIT in 13 cell subsets; (**E**) Flow cytometry analysis of the expression ratio of CD200R1 in 13 cell subsets; (**F**) Flow cytometry analysis of the expression ratio of LAG3 in 13 cell subsets; (**G**) Flow cytometry analysis of the expression ratio of CD160 in 13 cell subsets; (**H**) Flow cytometry analysis of the expression ratio of BTLA in 13 cell subsets; (**I**) Flow cytometry analysis of the expression ratio of LAIR1 in 13 cell subsets; (**J**) Flow cytometry analysis of the expression ratio of LILRB4 in 13 cell subsets.

**Figure 5:**
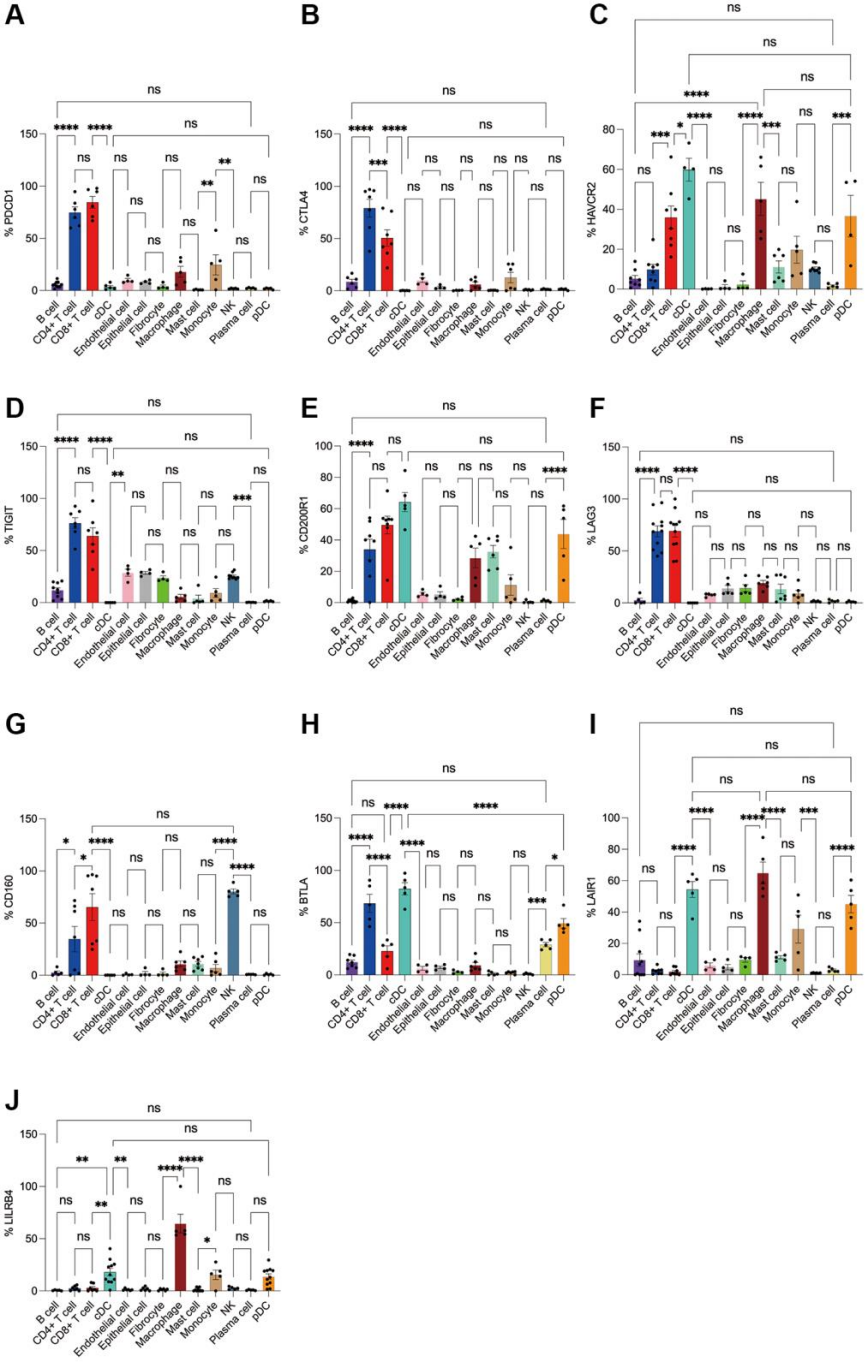
**Flow cytometry analysis of the differences in the expression proportions of 10 immune checkpoint receptors in 13 cell subsets in human colorectal cancer.** (**A**) Flow cytometry analysis of the expression ratio of PD-1 in 13 cell subsets; (**B**) Flow cytometry analysis of the expression ratio of CTLA4 in 13 cell subsets; (**C**) Flow cytometry analysis of the expression ratio of HAVCR2 in 13 cell subsets; (**D**) Flow cytometry analysis of the expression ratio of TIGIT in 13 cell subsets; (**E**) Flow cytometry analysis of the expression ratio of CD200R1 in 13 cell subsets; (**F**) Flow cytometry analysis of the expression ratio of LAG3 in 13 cell subsets; (**G**) Flow cytometry analysis of the expression ratio of CD160 in 13 cell subsets; (**H**) Flow cytometry analysis of the expression ratio of BTLA in 13 cell subsets; (**I**) Flow cytometry analysis of the expression ratio of LAIR1 in 13 cell subsets; (**J**) Flow cytometry analysis of the expression ratio of LILRB4 in 13 cell subsets.

**Figure 6:**
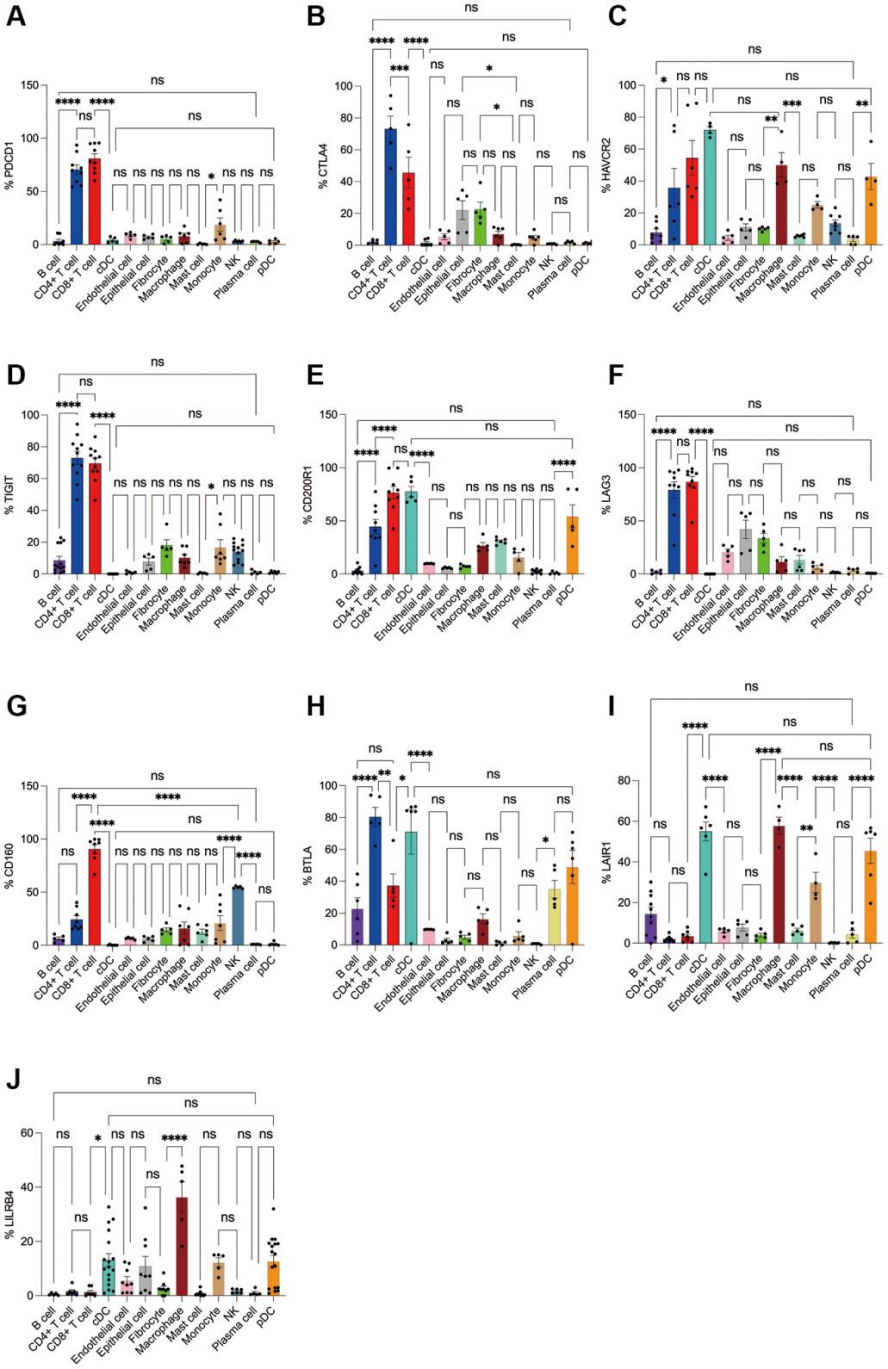
**Flow cytometry analysis of the differences in the expression proportions of 10 immune checkpoint receptors in 13 cell subsets in human lung cancer.** (**A**) Flow cytometry analysis of the expression ratio of PD-1 in 13 cell subsets; (**B**) Flow cytometry analysis of the expression ratio of CTLA4 in 13 cell subsets; (**C**) Flow cytometry analysis of the expression ratio of HAVCR2 in 13 cell subsets; (**D**) Flow cytometry analysis of the expression ratio of TIGIT in 13 cell subsets; (**E**) Flow cytometry analysis of the expression ratio of CD200R1 in 13 cell subsets; (**F**) Flow cytometry analysis of the expression ratio of LAG3 in 13 cell subsets; (**G**) Flow cytometry analysis of the expression ratio of CD160 in 13 cell subsets; (**H**) Flow cytometry analysis of the expression ratio of BTLA in 13 cell subsets; (**I**) Flow cytometry analysis of the expression ratio of LAIR1 in 13 cell subsets; (**J**) Flow cytometry analysis of the expression ratio of LILRB4 in 13 cell subsets.

Subsequently, we applied flow cytometry to validate in fresh samples of human breast cancer, colorectal cancer, and lung cancer. We can see that PD-1 is most expressed in CD4+ T cells and CD8+ T cells among all three tumors, and there is no difference between the two groups, followed by monocytes, although no difference is observed in breast cancer (Figures 4A, 5A, 6A). For CTLA4, CD4+ T cells were the most expressed, significantly higher than the second CD8+ T cells, and the expression of the rest of the cells was low, but epithelial cells and fibroblasts in lung cancer also expressed CTLA4 higher, which was not observed in breast cancer and colorectal cancer (Figures 4B, 5B, 6B). In addition, pDCs and CD8+ T cells were also found to express HAVCR2 in high expression and CD4+ T cells in lung cancer, while CD4+ T cells in breast cancer and colorectal cancer expressed HAVCR2 significantly lower than CD8+ T cells (Figures 4C, 5C, 6C). TIGIT was highly expressed on CD4+ T cells and CD8+ T cells in all three tumors, moderately expressed on NK cells in colorectal cancer, and moderately expressed in monocytes in lung cancer (Figures 4D, 5D, 6D). CD200R1 had the highest expression on cDCs, followed by CD8+ T cells, and moderate expression on CD4+ T cells, macrophages, and mast cells, which was consistent with bioinformatics analysis, except that pDC cells were also highly CD200R1 expressed by flow cytometry (Figures 4E, 5E, 6E). Similarly, flow cytometry results showed that LAG3 was highly expressed in CD4+ T cells and CD8+ T cells, with the remaining cell subsets being low or not expressed (Figures 4F, 5F, 6F). The results of CD160 were similar to the results of Bioxin, with high expression on CD8+ T cells and NK cells, in addition to CD4+ T cells in colorectal cancer, which was not observed in breast and lung cancer (Figures 4G, 5G, 6G). BTLA was found to be significantly increased in CD4+ T cells, cDCs, plasma cells, and pDCs. In contrast to bioinformatics analysis, flow cytometry analysis showed that CD8+ T cells expressed BTLA higher than B cells in breast cancer, and this trend was observed in colorectal cancer and lung cancer but no significant difference was observed (Figures 4H, 5H, 6H). In addition to the high expression of LAIR1 in macrophages, flow cytometry also found that cDCs and pDCs were also highly expressed in cDC and pDC cells in three tumors, and monocytes from colorectal and lung cancers were also found to express LAIR1 (Figures 4I, 5I, 6I). LILRB4 was most expressed in macrophages and moderately expressed in cDCs, and monocytes and pDCs were also found to express LILRB4 higher than most cell subsets (Figures 4J, 5J, 6J).

### Expression levels of immune checkpoint ligands in pan-cancer

**Figure 7:**
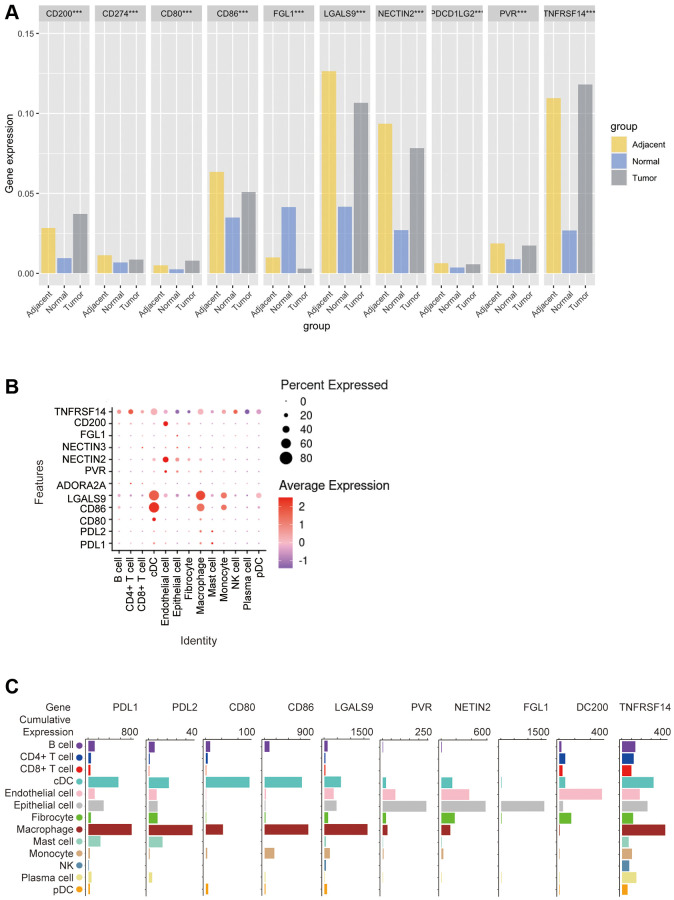
**Bioinformatics analysis of the distribution of 10 immune check site ligands on tissues and cells.** (**A**) Expression ratios of 10 immune checkpoint ligands in tumors, blood, spleen, lung, muscle, and bone marrow tissues; (**B**) Bioinformatics analysis of the expression of 10 immune checkpoint ligands in 13 cell subsets showed a heat map of the normalized markers for each cluster, with data representing the entire tumor; (**C**) Bioinformatics analysis of ligands expression at 10 immune checkpoints, histogram showing high and low expression of each receptor on different cell subsets, and data representing the entire tumor.

Next, we used the GEO database to analyze the expression of these 10 ICLs in tumor, adjacent and normal tissues. The results showed that CD200, CD80 and TNFRSF14 were the highest in tumor tissues, CD274 (PDL1), CD86, LGALS9, NECTIN2, PDL2, PVR were lower than those in adjacent tissues but higher than those in normal tissues, and FGL1 was the lowest in tumor tissues. It is important to note that these ligands are all least expressed in normal tissues, with the exception of FGL1, which is indeed the highest expressed in normal tissues. (Kruskal-Wallis test, *p* < 0.001) (Figure 7A).

In order to explore the correlation between ICLs expression and specific types of cell infiltration in the tumor immune microenvironment, the relative abundance of 13 cell types was inferred by gene set variant analysis (GSVA) based on the ICLs expression profile on TISIDB. The results showed that the expression of PDL1 and PDL2 was closely related to cDCs, macrophages and mast cells in TCGA tumors. The expression of CD80 and CD86 was closely related to cDCs and macrophages. LGALS9 expression was closely related to cDCs, macrophages and monocytes, ADORA2A and NECTIN3 expressions were not correlated with each cell subset, PVR expression was closely related to endothelial cells and epithelial cells, NECTIN2 and FGL1 expression were closely related to epithelial cells, CD200 expression was closely related to endothelial cells, and TNFRSF14 expression was closely related to cDCs, epithelial cells and macrophages (Figure 7B).

In addition, the correlation between the expression of these 10 ICLs and the gene expression of 13 cell subsets was again analyzed using Barplot. The results showed that the expression trends of PDL1 and PDL2 were similar in n TCGA tumors, with the highest expression levels on macrophages, and the expression levels in cDC, epithelial cells, The expression trend of CD86 was similar to that of CD80, which was highly expressed in both cDCs and macrophages, except that CD86 was most expressed in macrophages, while CD80 was highest in cDC cells, and CD86 was moderately expressed in mast cells without CD80, and LGALS9 was highly expressed in cDCs and macrophages, and was highly expressed in cDCs, CD200 was most expressed on endothelial cells and epithelial cells, followed by fibroblasts, and TNFRSF14 was most expressed on macrophages and moderately on cDCs, epithelial cells, endothelial cells, and B cells (Figure 7C).

**Figure 8:**
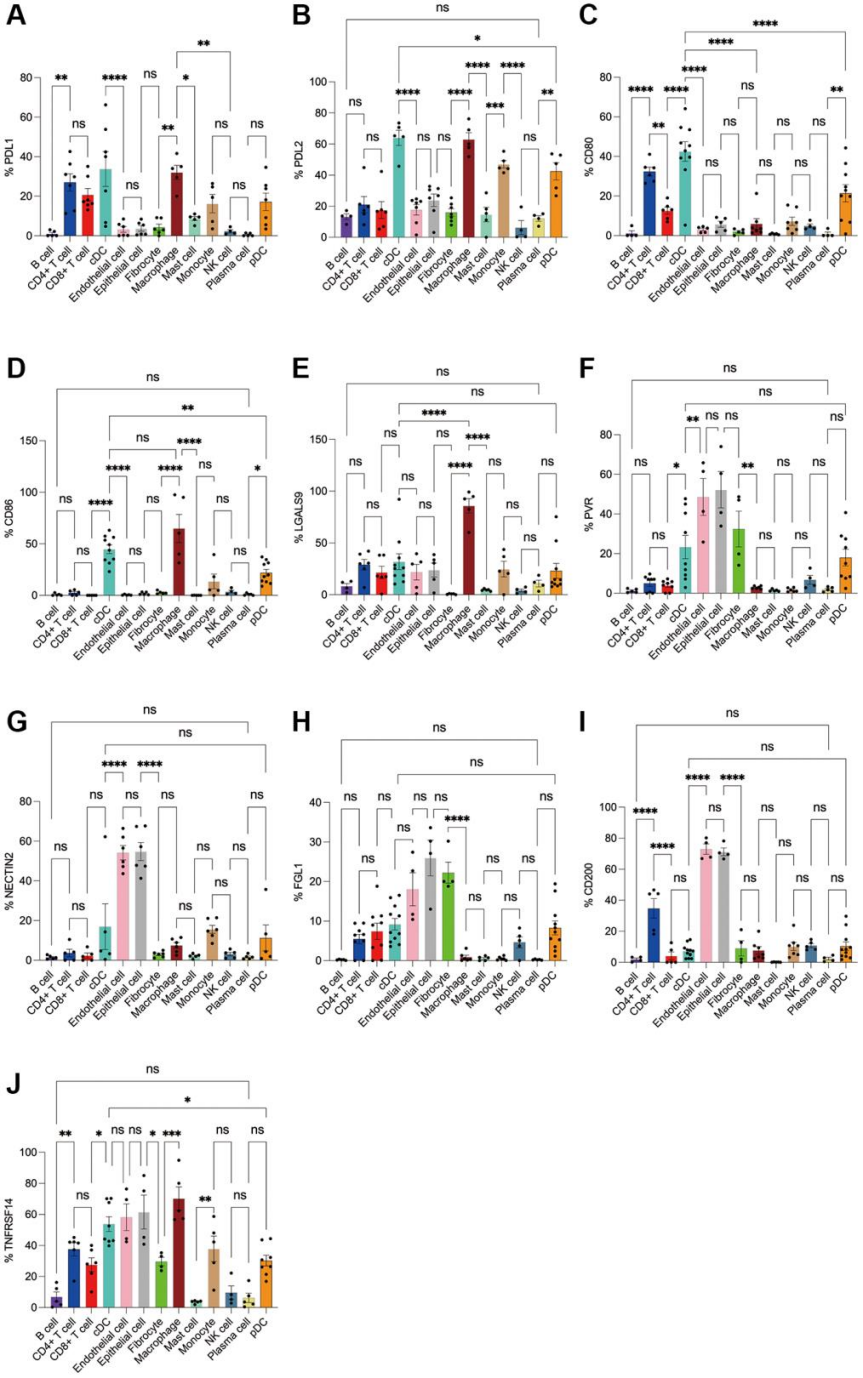
**Flow cytometry analysis of the differences in the expression proportions of 10 immune checkpoint ligands in 13 cell subsets in human breast cancer.** (**A**) Flow cytometry analysis of the expression ratio of PDL1 in 13 cell subsets; (**B**) Flow cytometry analysis of the expression ratio of PDL2 in 13 cell subsets; (**C**) Flow cytometry analysis of the expression ratio of CD80 in 13 cell subsets; (**D**) Flow cytometry analysis of the expression ratio of CD86 in 13 cell subsets; (**E**) Flow cytometry analysis of the expression ratio of LGALS9 in 13 cell subsets; (**F**) Flow cytometry analysis of the expression ratio of PVR in 13 cell subsets; (**G**) Flow cytometry analysis of the expression ratio of NECTIN2 in 13 cell subsets; (**H**) Flow cytometry analysis of the expression ratio of FGL1 in 13 cell subsets; (**I**) Flow cytometry analysis of the expression ratio of CD200 in 13 cell subsets; (**J**) Flow cytometry analysis of the expression ratio of TNFRSF14 in 13 cell subsets.

**Figure 9:**
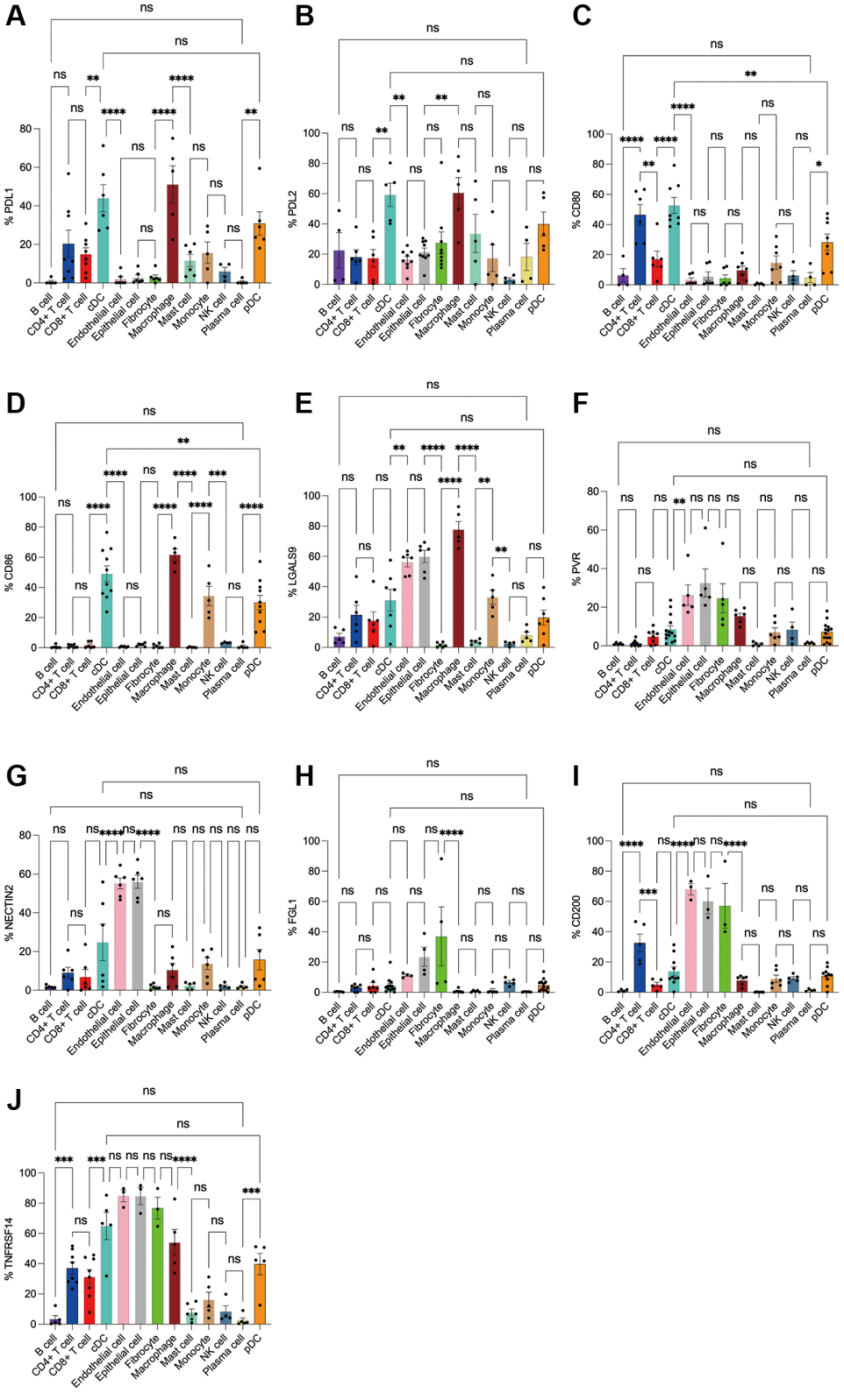
**Flow cytometry analysis of the differences in the expression proportions of 10 immune checkpoint ligands in 13 cell subsets in human colorectal cancer.** (**A**) Flow cytometry analysis of the expression ratio of PDL1 in 13 cell subsets; (**B**) Flow cytometry analysis of the expression ratio of PDL2 in 13 cell subsets; (**C**) Flow cytometry analysis of the expression ratio of CD80 in 13 cell subsets; (**D**) Flow cytometry analysis of the expression ratio of CD86 in 13 cell subsets; (**E**) Flow cytometry analysis of the expression ratio of LGALS9 in 13 cell subsets; (**F**) Flow cytometry analysis of the expression ratio of PVR in 13 cell subsets; (**G**) Flow cytometry analysis of the expression ratio of NECTIN2 in 13 cell subsets; (**H**) Flow cytometry analysis of the expression ratio of FGL1 in 13 cell subsets; (**I**) Flow cytometry analysis of the expression ratio of CD200 in 13 cell subsets; (**J**) Flow cytometry analysis of the expression ratio of TNFRSF14 in 13 cell subsets.

**Figure 10:**
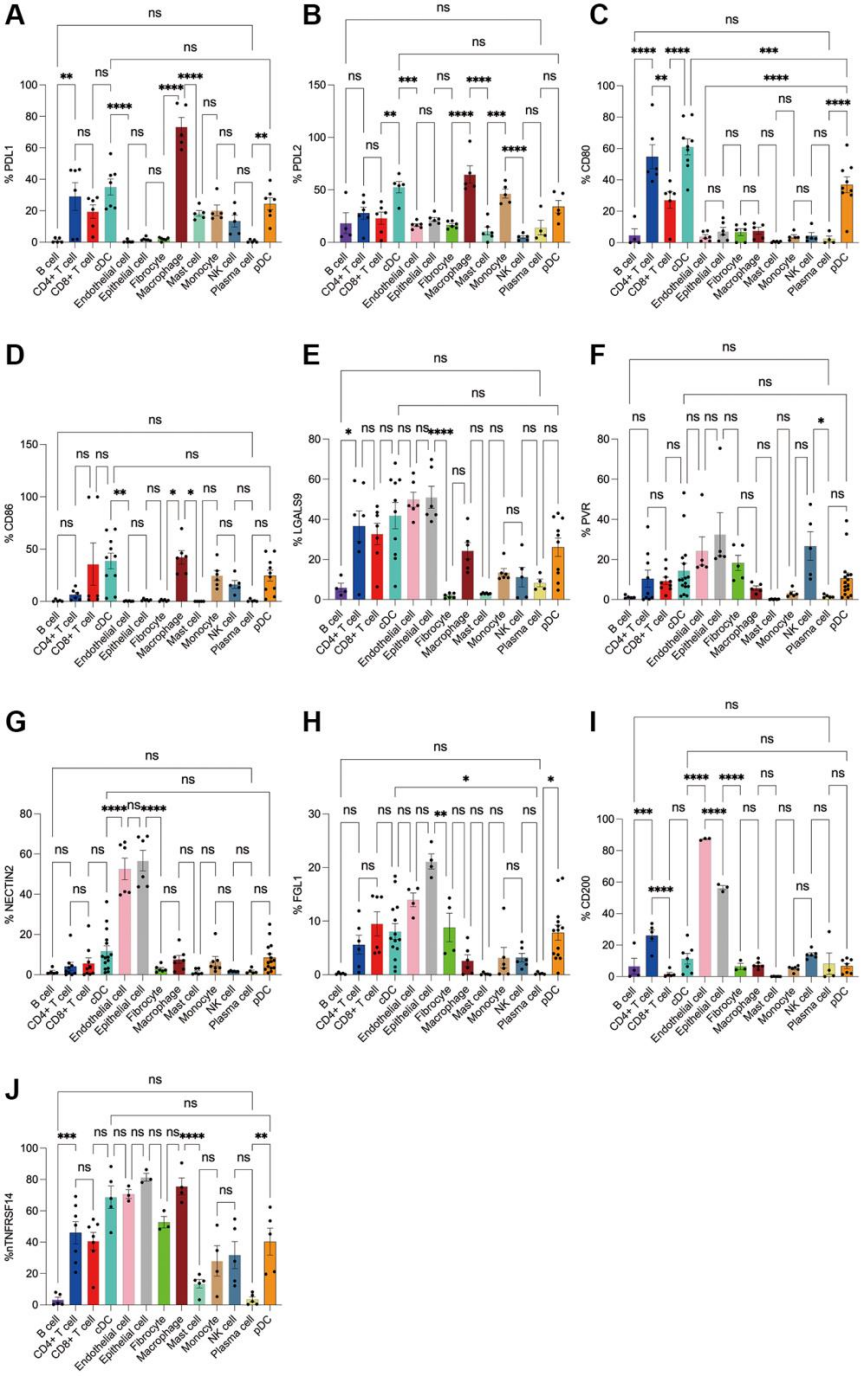
**Flow cytometry analysis of the differences in the expression proportions of 10 immune checkpoint ligands in 13 cell subsets in human lung cancer.** (**A**) Flow cytometry analysis of the expression ratio of PDL1 in 13 cell subsets; (**B**) Flow cytometry analysis of the expression ratio of PDL2 in 13 cell subsets; (**C**) Flow cytometry analysis of the expression ratio of CD80 in 13 cell subsets; (**D**) Flow cytometry analysis of the expression ratio of CD86 in 13 cell subsets; (**E**) Flow cytometry analysis of the expression ratio of LGALS9 in 13 cell subsets; (**F**) Flow cytometry analysis of the expression ratio of PVR in 13 cell subsets; (**G**) Flow cytometry analysis of the expression ratio of NECTIN2 in 13 cell subsets; (**H**) Flow cytometry analysis of the expression ratio of FGL1 in 13 cell subsets; (**I**) Flow cytometry analysis of the expression ratio of CD200 in 13 cell subsets; (**J**) Flow cytometry analysis of the expression ratio of TNFRSF14 in 13 cell subsets.

Subsequently, we applied flow cytometry to validate in fresh samples of human breast cancer, colorectal cancer, and lung cancer. Among the three tumors, PDL1 and PDL2 were the highest expressed in macrophages, followed by cDCs, and mast cells were expressed in low amounts. Unlike in the bioinformatics results, CD4+ T cells, CD8+ T cells, monocytes, and pDC cells were found to express PDL1 and PDL2 in moderate amounts by flow cytometry (Figures 8A, 8B, 9A, 9B, 10A, 10B). For CD80, in addition to the high expression of cDCs and the moderate expression of macrophages, high expression of CD4+ T cells, moderate expression of CD8+ T cells and pDCs were also found to be significantly higher than those of other cell subsets (Figures 8C, 9C, 10C). The results of CD86 showed high expression on cDCs and macrophages, which were consistent with the results of bioinformatics analysis. In addition, pDCs were also found to moderately express CD86, and monocytes in colorectal cancer also moderately expressed CD86 (Figures 8D, 9D, 10D). The expression of LGALS9 cell subsets in the three tumors was quite different, with macrophages being the highest in breast cancer and lower in the rest of the cell subsets, and macrophages, epithelial cells, endothelial cells, and monocytes were all expressed more in colorectal cancer, while macrophages were only moderately expressed in lung cancer, and epithelial cells, endothelial cells, cDCs, CD8+ T cells, CD4+ T cells, and pDCs were all expressed higher (Figures 8E, 9E, 10E). PVR showed high expression on endothelial cells, epithelial cells, fibroblasts, and cDCs in all three tumors, but only in breast cancer showed significant differences from other cell subsets, and unlike the bioinformatics results, pDCs also expressed moderate amounts of PVR (Figures 8F, 9F, 10F). The results of the NECTIN2 were also similar to the results of Bioxin, with the highest expression in epithelial cells and endothelial cells, and there was no difference between the two cells (Figures 8G, 9G, 10G). FGL1 was found to be most expressed in epithelial cells of breast and colorectal cancer, followed by fibroblasts, and there was a significant difference in lung cancer (Figures 8H, 10H). In addition to this, we also found differences from bioinformatics analysis, which found that FGL1 was highly expressed in endothelial cells, cDCs, CD8+ T cells, CD4+ T cells, and pDCs in breast and lung cancers, while only fibroblasts and epithelial cells were moderately expressed in colorectal cells (Figures 8H, 9H, 10H). In addition to validating the high expression of CD200 in endothelial cells in bioletters, flow cytometry also found that epithelial cells and CD4+ T cells also expressed CD200 in three tumors, and fibroblasts in colorectal cancer were highly expressed CD200 (Figures 8I, 9I, 10I). The TNFRSF14 of breast and lung cancer is most expressed in macrophages, while in colorectal cancer the highest expression is in endothelial, epithelial, and fibroblasts, and macrophages are only moderately expressed (Figures 8J, 9J, 10J). CD4+ T cells, CD8+ T cells, monocytes, and pDCs were moderately expressed in the three tumors, which was TNFRSF14 higher than the proportion shown in the bioinformatics results. (Figures 8J, 9J, 10J).

### Correlation analysis and prognostic value of immune checkpoint expression in pan-cancer

**Figure 11:**
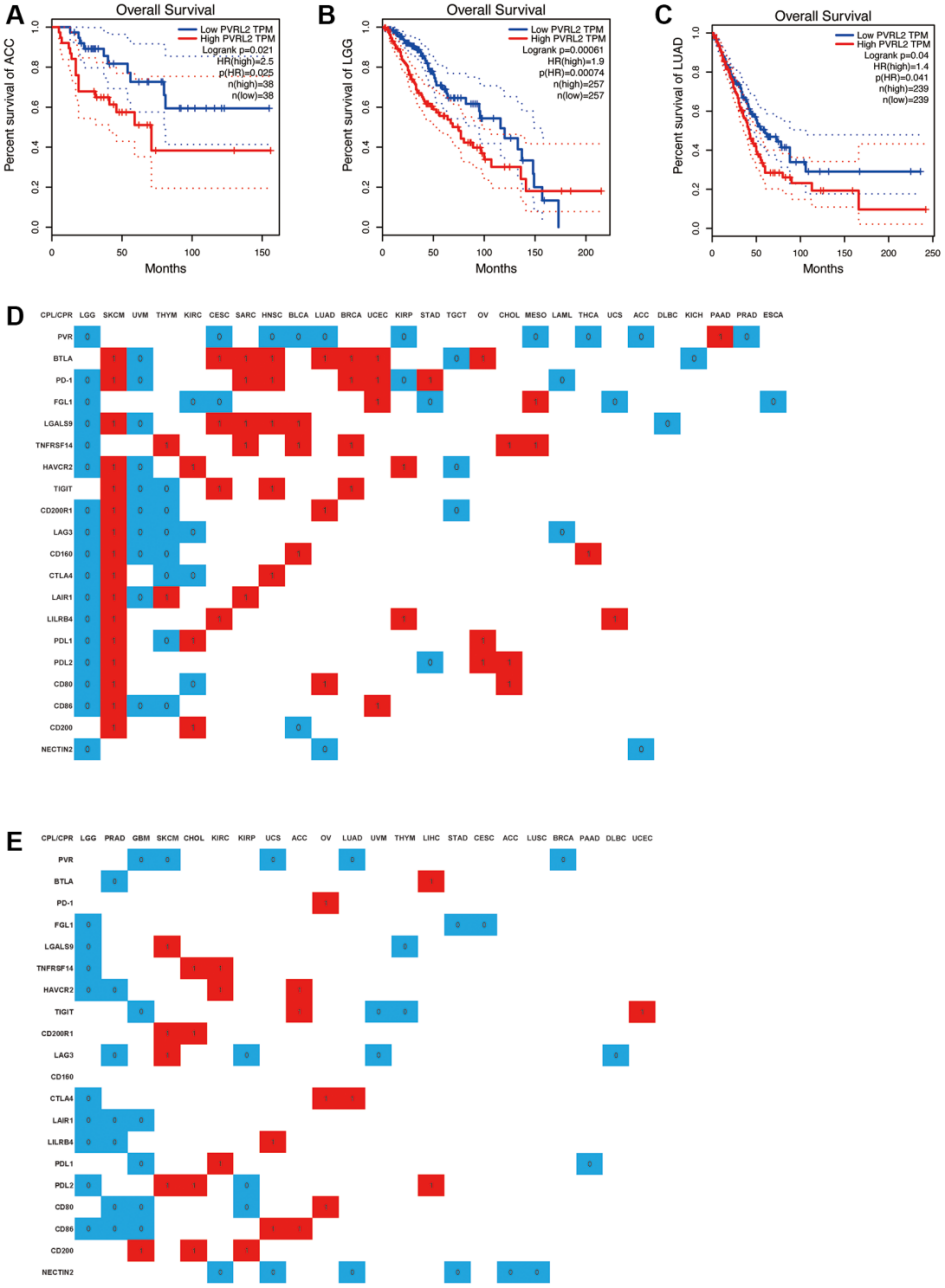
**A significant correlation between immune checkpoint receptor/ligand expression and pan-cancer prognostic value is dependent on DriverDBv3.** Expression of NECTIN2 was associated with overall survival (OS) in ACC (**A**), LGG (**B**), and LUAD (**C**); (**D**) Correlation between the expression of 20 immune checkpoint receptors/ligands and OS of various tumors, red: positive correlation, *P* < 0.05, blue: negative correlation, *P* < 0.05, white: no significant difference, *P* > 0.05; (**E**) Correlation between the expression of 20 immune checkpoint receptors/ligands and PFS of various tumors, red: positive correlation, *P* < 0.05, blue: negative correlation, *P* < 0.05, white: no significant difference, *P* > 0.05.

Use DriverDBv3 to study the relationship between the expression of immune checkpoint receptors and ligands and the clinical characteristics of various tumors, including overall survival (OS), progression free survival (PFS), and evaluate the prognostic value of the expression of various immune checkpoint receptors and ligands in different tumors. Lower NECTIN2 expression levels were associated with longer OS (HR > 1) in ACC, LGG, and LUAD (Figure 11A–11D), and in KIRC, UCS, LUAD, STAD, ACC, and LUSC, lower NECTIN2 expression levels were associated with longer PFS (HR > 1) (Figure 11A–11D; Supplementary Figures 1–20). The expression of CD200 was positively correlated with the OS of SKCM and KIRC, and the PFS of GBM, CHOL, and KIRP, but negatively correlated with the OS of BLCA; the expression of CD86 was positively correlated with the OS of SKCM, UCEC, and the PFS of UCS and ACC, but was negatively correlated with the OS of LGG, UVM, THYM’s OS, LGG, PRAD, and GBM’s PFS were negatively correlated; CD80 expression was positively correlated with SKCM, LUAD, CHOL’s OS, and OV’s PFS, while it was positively correlated with LGG, KIRC’s OS, PRAD, GBM, and KIRP’s PFS was negatively correlated; the expression of PDL2 was positively correlated with the OS of SKCM, OV, CHOL, and the PFS of SKCM, CHOL, and LIHC, and negatively correlated with the OS of LGG and STAD, and the PFS of LGG and KIRP; the expression of PDL1 was positively correlated with SKCM, KIRC, OV’s OS, KIRC’s PFS were positively correlated, and negatively correlated with LGG, THYM’s OS, and GBM’s PFS; LILRB4 expression was positively correlated with SKCM, CESC, KIRP, UCS’s OS, UCS’s PFS, and LGG’s OS. The PFS of LGG and PRAD were negatively correlated; the expression of LAIR1 was positively correlated with the OS of SKCM, THYM, and SARC, and negatively correlated with the OS of LGG and UVM, and the PFS of LGG, PRAD, and GBM; the expression of CTLA4 was positively correlated with the OS of SKCM and HNSC, the PFS of OV and LUAD was positively correlated, and negatively correlated with the OS of LGG, THYM, KIRC, and the PFS of LGG; the expression of CD160 was positively correlated with the OS of SKCM, BLCA, and THCA, and negatively correlated with the OS of LGG, UVM, and THYM, and there is no significant difference in the PFS analysis of cancer types; the expression of LAG3 is positively correlated with the OS and PFS of SKCM, and negatively correlated with the OS of LGG, UVM, THYM, KIRC, LAML, and the PFS of PRAD, KIRP, UVM, and DLBC. The expression of CD200R1 was positively correlated with the OS of SKCM and LUAD, the PFS of SKCM and CHOL, and negatively correlated with the OS of LGG, UVM, THYM, and TGCT; the expression of TIGIT was positively correlated with the OS of SKCM, CESC, HNSC, BRCA, ACC. The PFS of UCEC was positively correlated with the OS of UVM and THYM, and negatively correlated with the PFS of GBM, UVM, and THYM; the expression of HAVCR2 was positively correlated with the OS of SKCM, KIRC, and KIRP, and the PFS of KIRC and ACC, and negatively correlated with the PFS of LGG, UVM, and TGCT. The OS and PFS of LGG and PRAD were negatively correlated; the expression of TNFRSF14 was positively correlated with the OS of THYM, SARC, BLCA, BRCA, CHOL, MESO, and the PFS of CHOL and KIRC, and negatively correlated with the OS and PFS of LGG; the expression of LGALS9 was positively correlated with the OS of SKCM, CESC, SARC, HNSC, and BLCA, and the PFS of SKCM, and negatively correlated with the OS of LGG, UVM, DLBC, and the PFS of LGG and THYM; the expression of FGL1 was positively correlated with the OS of UCEC and MESO. There was a negative correlation with the OS of LGG, KIRC, CESC, STAD, UCS, and ESCA, and the PFS of LGG, STAD, and CESC; the expression of PD-1 was positively correlated with the OS of SKCM, SARC, HNSC, BRCA, UCEC, STAD, and the PFS of OV and negatively correlated with OS of LGG, UVM, KIRP, and LAML; BTLA expression was positively correlated with OS of SKCM, CESC, SARC, HNSC, LUAD, BRCA, UCEC, OV, and PFS of LIHC, and UVM, TGCT, KICH OS, PFS of PRAD were negatively correlated; PVR expression was positively correlated with OS of PAAD, and with OS of LGG, CESC, HNSC, BLCA, LUAD, KIRP, MESO, THCA, ACC, PRAD, GBM, SKCM, UCS, LUAD, PFS of BRCA were negatively correlated (Figure 11D, 11E).

In addition, we found that all CPR/CPL expressions with significant differences in LGG, CHOL, and OV were negatively correlated with OS and PFS, and all CPR/CPL expressions with significant differences in SKCM, SARC, BRCA, and UCEC were negatively correlated with OS and PFS. All are positively correlated with OS. All CPR/CPL expressions with significant differences in UVM, TGCT, LAML, and ACC are negatively correlated with OS. All CPR/CPL expressions with significant differences in ACC and LIHC are positively correlated with PFS, all CPR/CPL expressions with significant differences in PRAD, UVM, THYM, and STAD were negatively correlated with PFS.

## DISCUSSION

Triggering suppressive immune checkpoints in TILs hampers cancer immune surveillance. Additionally, cancer cells elevate immune checkpoints due to local inflammation, fostering tumor growth and aggressiveness. Thus, ICBs serve not only to restore depleted/dysfunctional TILs but also to directly impact tumor cells. In our study, we investigated the expression patterns of new and old immune examination site receptors and ligands, including PD-1 and CTLA4, as well as 13 immune microenvironment cells in TCGA pan-cancer. We found that high expression of PVR and NECTIN2 predicts poor prognosis in a variety of cancers. Coincidentally, they are both ligands for TIGIT, and the expression of PVR and NECTIN2 is strongly correlated with epithelial and endothelial cells, and are more expressed in tumor tissues than in normal tissues. These results suggest that the expression of PVR and NECTIN2 is associated with the clinical features of a variety of tumors and can be used as prognostic predictors of LGG, LUAD, and ACC.

It is well known that PVR is associated with the tumor immune microenvironment [53]. PVR is considered to be a modulator of the immune response in cancers such as LUAD, colorectal cancer, melanoma, etc., [54–57]. NECTIN2 also gives new expectations in cancers such as HCC, BRCA, PRAD [58–60]. Our analysis revealed the correlation between PVR and NECTIN2 expression and the tumor immune microenvironment or tumor microenvironment in cancer, and further solidified the critical role of PVR and NECTIN2 in tumor immune regulation. Subsequently, our flow cytometry results revealed that PVR, NECTIN2 epithelial cells, endothelial cells, fibroblasts, cDCs, and pDCs concentrated in tumor tissue, as expected. Other reports suggest that PVR, NECTIN2 are expressed on epithelial and endothelial cells. Our findings confirm these results to some extent. In addition, the latest studies have pointed out that in most tumors, the expression of PVR and NECTIN2 is positively correlated with PD-L1, B7-H3, LGALS9 and CD80/CD86 [61–63]. Upregulation of immune checkpoints and immunosuppressive molecules (e.g., PD-L1, B7-H3, LGALS9 and CD80/CD86) has been described as one of the hallmarks of T cell depletion and suggests a state of T cell dysfunction [64, 65]. T cell depletion status has been reported to serve as a drug guide for immunotherapy [65]. Based on these findings, we can speculate that PVR and NECTIN2 may predict T cell status based on our results and may become molecular markers for immunotherapy. It is worth noting that PVR, NECTIN2 have a low correlation with immune checkpoint receptors in tumors, which may be due to the almost non-existent expression of PVR and NECTIN2 on immune cells (Figure 7) and explain the beneficial role of low expression of PVR, NECTIN2 in tumor survival assays.

LGG is a common malignant primary tumor in the central nervous system, and most patients will eventually develop a highly aggressive glioma despite comprehensive conventional treatment. Notably, our results suggest that OS and PFS of LGG are inversely correlated in the expression of various ICRs/ICLs. Recent studies have shown that LGGs in the poor prognostic subtype are characterized by high mutational burden, high frequency of copy number changes, and high levels of tumor-infiltrating lymphocyte and immune checkpoint genes [66]. However, the study was limited to PD-1/PD-L1/CTLA-4. In addition, LGG has a unique immunosuppressive tumor immune microenvironment compared to IDH-wild-type HGG and brain metastases, but there is still a lack of awareness of the LGG immune microenvironment, potential biological pathways, and impact on immunotherapy [67].

To combat melanoma, surgery stands as the primary approach for treating localized instances of this formidable skin cancer. Yet, even after excising the tumor, the potential for both local and distant recurrence looms, especially in cases of thicker or ulcerated tumors, or when lymph nodes are affected [68]. Immunotherapy utilizing immune checkpoint inhibitors (ICIs) directed at PD-1, PD-L-1, or CTLA-4 has become a prominent treatment strategy for SKCM. Additionally, innovative approaches such as anti-LAG-3 ICIs, adoptive cell therapies, intratumoral immunotherapies, and cancer vaccines are under development to address drug resistance and enhance patient outcomes [69].

UVM stands as the prevalent primary intraocular malignancy among adults, which exhibits less responsiveness to chemotherapy or immune checkpoint inhibitors compared to SKCM [70]. There is a suggestion that immune cells infiltrating UVM encompass a variety of cell types, including CD8+ T cells that primarily express the checkpoint marker LAG3 rather than PD1 or CTLA4. This proposes LAG3 as a potential target for immune checkpoint blockade in high-risk UVM patients [71].

PRAD ranks as the primary cause of mortality among men [72], and inherently exhibits resistance to ICBs [73], and neither monotherapy targeting anti-PD-1/PD-L1 nor anti-CTLA-4 demonstrates a significant impact on the overall survival of prostate cancer patients [74, 75]. Resistance to ICBs in PRAD has been associated with intrinsic mechanisms within tumor cells as well as the limited presence of immune infiltrates dominated by macrophages [76]. In PRAD, tumor-specific CD4+ and CD8+ T cells exhibit rapid upregulation of LAG-3 upon encountering antigens *in vivo*. Treatment with anti-LAG-3 monoclonal antibodies enhances both the number and effector function of tumor-specific CD8+ T cells in TRAMP mice, thereby slowing tumor growth [77]. Moreover, Tregs within human PRAD lesions demonstrate upregulation of CTLA-4 and LAG-3 [78]. However, recent data have shown low expression of LAG-3 in infiltrative PRAD lesions, which challenges the aforementioned finding [79]. Further research is necessary to elucidate the precise role of LAG-3 in T cell depletion and/or Treg function in PRAD. Currently, a clinical trial is underway to investigate the efficacy of an anti-LAG-3 monoclonal antibody in combination with anti-PD-1 for the treatment of castration-resistant PRAD (ClinicalTrials.gov identifier: NCT03365791). Data regarding TIM-3 in PRAD patients are conflicting. One study indicates that elevated TIM-3 expression on PRAD cells predicts shorter recurrence-free survival and progression-free survival in chemotherapy- and radiotherapy-naïve PRAD patients [80]. Conversely, other studies have identified negative TIM-3 expression as an independent prognostic factor for a poor prognosis in advanced metastatic PRAD [81].

## MATERIALS AND METHODS

### Human samples

Fresh samples of 286 cases of breast cancer, 302 cases of colorectal cancer, and 308 samples of lung cancer (see Table 1 for clinical data of patients) were collected from the operating room of our hospital to isolate immune cells and stromal cells.

**Table 1:** Characteristics of clinicopathological information in patients with colorectal, breast, and lung cancer.

	**All patients**	**Colorectal cancer**	**Breast cancer**	**Lung cancer**
Number	896	302	286	308
Age (range)	49.38 (25–70)	48.81 (29–70)	47.26 (25–68)	51.91 (35–70)
Male/Female	367/	171/131	2/284	194/114
Tumor size		≤5 cm:	≤2 cm:	≤10 cm:
		>5 cm:	>2 cm:	>10 cm:
TNM stage, number (%)				
I		61 (20.20)	86 (30.07)	47 (15.26)
II		93 (30.79)	115 (40.21)	78 (25.32)
III		90 (29.80)	57 (19.93)	93 (30.19)
IV		58 (19.21)	28 (9.90)	90 (29.22)
Vascular invasion		91 (30.13)	64 (22.38)	137 (44.48)
Lymphatic invasion		99 (32.78)	87 (30.42)	123 (39.94)

TNM stage is referred to AJCC cancer staging manual (8th version).

### Specimen collection

Within 30 minutes of separation of the surgical specimens, the intestinal lumen was longitudinally dissected in the sterile environment of the operating room, rinsed with sterile saline, and an appropriate amount of colorectal cancer, paired cancer, and normal tissue specimens were cut with a sterile scalpel. Masses should be selected to avoid necrotic or purulent tissue. Paracancerous tissue is defined as a 2–3 cm opening on the edge of the lump. Paired normal tissue is defined as the colorectal mucosal epithelial tissue with normal margins (2–10 cm from the tumor). The obtained tissue specimens were placed in 10× bispecific antibody 1640 medium and quickly transferred to the laboratory or −80°C cryogenic freezer for storage in a cryogenic container.

### Primary cell separation

Trim the tissue into a sterile six-well plate to remove necrosis, bruising, fat, and excess tissue. Specimen washing was performed: 10× bispecific antibody + 40 μg/mL gentamicin PBS 25 mL in a 50 mL sterile centrifuge tube were washed 3 times. Then, 10× bispecific antibody + 40 μg/mL gentamicin 10% FBS 1640 were washed in a 50 mL sterile centrifuge tube at 40–60 rpm for 15 min × 3 times, each microscopic examination. For each of these steps, the sterile centrifuge tube needs to be replaced. Tissue digestion is followed: 1–2 mm of tissue is first minced with sterile ophthalmic scissors. (Optionally, a 1 mM dithiothreitol (DTT) solution in PBS containing tissue equivalent to 6 times the amount of tissue pellet was added and incubated at 37°C for 30 min.) Then centrifuge at 800 rpm for 5 min and discard the supernatant, followed by 1:6 digest (collagenase 41 mg/mL, hyaluronidase 20 μg/mL) at 37°C for 2 h with vigorous shaking for 30 sec every 30 minutes. It was then filtered with a 40 μm filter. Finally, lymphocyte pre-enrichment (optional): the first step is 800 rpm low-speed centrifugation to remove debris. In the second step, resuspend the cells in complete medium (lymphocytes pre-enriched with 70–30% percoll centrifugation for 25 min). The third step, PBS wash 2 times. Finally, the pellet is resuspended in complete medium overnight or for further testing.

### Flow cytometry

Flow cytometry is divided into two parts, the first is extracellular staining: the first step is to collect cells, 2500 rpm for 5 min, and discard the supernatant; stain with buffer 1 mL at 2500 rpm, wash cells for 5 min× 1 time. In the second step, discard the supernatant and resuspend the cells with 50 μL of buffer. In the third step, add 2-5-10-20 μL of antibody, and incubate for 15 min at room temperature and in the dark. Finally, stain buffer 1 mL at 2500 rpm for 5 min, wash once, and carefully aspirate and discard the supernatant.

For the intracellular stained fraction: first collect the cells, 2,500 rpm for 5 min, and discard the supernatant. The cells were then resuspended in 250 μL of fixation and perforated at 4°C for 20 minutes. Then 1 mL wash buffer 3500 rpm was washed twice in 5 minutes. Cells are then resuspended in 50 μL of staining buffer. Antibodies were then added, 2-5-10-20 μL, protected from light, and incubated for 30 min. Then stain buffer 1 mL at 3500 rpm and wash once for 5 min. Finally, stain the cells with buffer 250 μL-500 μL and resuspend them for detection.

### Data collection

We downloaded a total of 1217 breast cancer patients’ gene expression profiles from The Cancer Genome Atlas (TCGA) based on the cancer genomics data analysis platform UCSC Xena (https://xena.ucsc.edu/, accessed on 17 June 2022) [33]. By integrating clinical data such as age, grade, survival status, and survival time, a total of 1069 cases of breast cancer patients were obtained. Then, the gene expression profiles (GSE20711) [34] and clinical information of 90 breast cancer patients were retrieved from the Gene Expression Omnibus (GEO) (https://www.ncbi.nlm.nih.gov/geo/ accessed on 3 July 2022). For the above datasets, we converted the Ensemble ID into a gene symbol and averaged the values when multiple probes were mapped to the same gene.

### Correlation of immune checkpoints expression to tumor clinical characteristics

GEPIA2 (http://gepia2.cancer-pku.cn/#index) was used to analyze the correlation between tumor stages and Siglec-9 mRNA expression using “major stage” and “log2 (TPM + 1) for log-scale”. The association between Siglec-9 mRNA expression and overall survival (OS), disease-specific survival (DSS), disease-free interval (DFI), and progression-free interval (PFI) depended on TCGA databases and was analyzed on the DriverDBv3 (http://driverdb.tms.cmu.edu.tw/) with mean of Siglec-9 expression as cutoff value. The online database UALCAN network (http://ualcan.path.uab.edu/) was also used to verify the OS analysis with default settings. Siglec-9 expression in diverse molecular subtypes was analyzed on The Integrated Repository Portal for Tumor-Immune System Interactions (TISIDB, http://cis.hku.hk/TISIDB/index.php).

### Correlation of immune checkpoints expression to immunological characteristics

TIMER (https://cistrome.shinyapps.io/timer/) and TIMER2.0 (http://timer.comp-genomics.org/) were used to assess the correlation between the Siglec-9 expression and immune cell infiltration for TCGA tumors and CGGA-LGG dataset with TIMER score and CIBERSORT score (Newman et al., 2015) using immune infiltrates query function and estimation function. The results of estimation were visualized by R package “psych”. TISIDB, a user-friendly web instrument to explore comprehensive investigation of tumor-associated immunity, was used to analyze the association between Siglec9 mRNA expression and tumor immune subtypes, and specific types of immune cell infiltration. We estimated Siglec-9 expression in diverse molecular subtypes, and immune subtypes involving C1 (wound healing), C2 (IFN-γ dominant), C3 (inflammatory), C4 (lymphocyte deplete), C5 (immunologically quiet), and C6 (TGF-β dominant) subtypes. Specific types of immune cell infiltration of 28 TIL types were inferred by using gene set variation analysis (GSVA) based on the Siglec-9 expression profile on TISIDB.

### Statistical analysis

The expression of ICRs and ICLs in different tissue was used by Kruskal–Wallis test, and between tumor tissues and normal tissues were estimated by *t*-test. Additionally, the expression of ICRs and ICLs in different grades of glioma was utilized by *t*-test and ANOVA test. Kaplan–Meier curves were visualized to compare the survival patients stratified based on different levels of expression of ICRs and ICLs. The relationship between ICRs and ICLs and TMB (tumor mutation burden), MSI (microsatellite instability), MMR gene mutation, immune checkpoints, DNMT, immune score, stromal score, ESTIMATE score, and immune cells was evaluated by Pearson and Spearman correlation analyses. *p* < 0.05 was recorded as statistically significant for all analyses unless otherwise specified.

### tSNE

Statistical analysis was conducted using RStudio (R version 4.1.2). The counts data was read using the CreateSeuratObject function from the Seurat package (Version 4.0.4) and the matadata was read using the read.table function. The identified clusters were visualized using the tSNE method. For subcluster analysis, similar procedures were used, including normalization, variably expressed feature selection, dimension reduction, and cluster identification. To annotate different clusters, the FindAllMarkers function was used to identify differential expression markers in the resulting clusters by the default non-parametric Wilcoxon rank sum test with Bonferroni correction. The top 3 markers between different clusters were visualized by the plot_genes_by_group function in monocle3 package (Version 1.0.0).

### Heatmap

Visualization of the proportion of each cell type was performed using the heatmap package (Version 1.0.12).

### Barplot

For 20 markers (PDCD1, CTLA4, HAVCR2, TIGIT, CD200R1, LAG3, CD160, BTLA, LAIR1, LILRB4, CD274, PDCD1LG2, CD80, CD86, LGALS9, PVR, NECTIN2, FGL1, CD200, TNFRSF14), the proportion of positive cells was calculated separately, and chi square test was used for inter group comparison between tumor, normal, and adjacent tissues. The barplots were visualized by the ggplot function in ggplot2 package (Version 3.3.5).

### Data availability statement

This work is based on a secondary analysis of publicly available datasets. Informed consent is not required. Data used and analyzed during the current study are available from UCSC Xena (https://xena.ucsc.edu) and GEO (https://www.ncbi.nlm.nih.gov/geo). The datasets generated and analyzed in this study may be obtained from the corresponding authors upon reasonable request.

## CONCLUSIONS

In summary, our investigation reveals associations between various immune checkpoint expressions and their correlation with pan-cancer as well as specific cellular components of the immune microenvironment. PVR and NECTIN2 expression serve as prognostic indicators in patients diagnosed with LGG, LUAD, ACC, and UCS. Across most tumor types, there exists a robust correlation between PVR and NECTIN2 expression and infiltration of epithelial cells. However, in LUAD and UCS, epithelial cells do not emerge as the primary cellular component, suggesting that PVR and NECTIN2 might hold greater significance in LGG and ACC. Notably, elevated levels of PVR and NECTIN2 are predictive of poorer prognosis and could potentially influence the progression of LGG and ACC through immune modulation and disruption of normal physiological functions. Overall, our findings propose PVR and NECTIN2 as promising biomarkers for prognostic assessment and evaluation of immune infiltration across various tumor types, particularly in LGG and ACC. Nevertheless, further experimental validation is essential to corroborate these findings.

## Supplementary Materials

Supplementary Figures

## References

[1] Li ZX, Zheng ZQ, Wei ZH, Zhang LL, Li F, Lin L, Liu RQ, Huang XD, Lv JW, Chen FP, He XJ, Guan JL, Kou J, et al. Comprehensive characterization of the alternative splicing landscape in head and neck squamous cell carcinoma reveals novel events associated with tumorigenesis and the immune microenvironment. Theranostics. 2019; 9:7648–65. 10.7150/thno.36585 31695792 PMC6831462

[2] Zhou R, Zhang J, Zeng D, Sun H, Rong X, Shi M, Bin J, Liao Y, Liao W. Immune cell infiltration as a biomarker for the diagnosis and prognosis of stage I-III colon cancer. Cancer Immunol Immunother. 2019; 68:433–42. 10.1007/s00262-018-2289-7 30564892 PMC6426802

[3] Xie P, Ma Y, Yu S, An R, He J, Zhang H. Development of an Immune-Related Prognostic Signature in Breast Cancer. Front Genet. 2020; 10:1390. 10.3389/fgene.2019.01390 32047513 PMC6997532

[4] Emens LA. Breast Cancer Immunotherapy: Facts and Hopes. Clin Cancer Res. 2018; 24:511–20. 10.1158/1078-0432.CCR-16-3001 28801472 PMC5796849

[5] Steven A, Fisher SA, Robinson BW. Immunotherapy for lung cancer. Respirology. 2016; 21:821–33. 10.1111/resp.12789 27101251

[6] Ma LJ, Feng FL, Dong LQ, Zhang Z, Duan M, Liu LZ, Shi JY, Yang LX, Wang ZC, Zhang S, Ding ZB, Ke AW, Cao Y, et al. Clinical significance of PD-1/PD-Ls gene amplification and overexpression in patients with hepatocellular carcinoma. Theranostics. 2018; 8:5690–702. 10.7150/thno.28742 30555574 PMC6276293

[7] Kennedy A, Waters E, Rowshanravan B, Hinze C, Williams C, Janman D, Fox TA, Booth C, Pesenacker AM, Halliday N, Soskic B, Kaur S, Qureshi OS, et al. Differences in CD80 and CD86 transendocytosis reveal CD86 as a key target for CTLA-4 immune regulation. Nat Immunol. 2022; 23:1365–78. 10.1038/s41590-022-01289-w 35999394 PMC9477731

[8] Qian W, Zhao M, Wang R, Li H. Fibrinogen-like protein 1 (FGL1): the next immune checkpoint target. J Hematol Oncol. 2021; 14:147. 10.1186/s13045-021-01161-8 34526102 PMC8444356

[9] Ming Q, Celias DP, Wu C, Cole AR, Singh S, Mason C, Dong S, Tran TH, Amarasinghe GK, Ruffell B, Luca VC. LAG3 ectodomain structure reveals functional interfaces for ligand and antibody recognition. Nat Immunol. 2022; 23:1031–41. 10.1038/s41590-022-01238-7 35761082 PMC10191176

[10] Lozano E, Mena MP, Díaz T, Martin-Antonio B, León S, Rodríguez-Lobato LG, Oliver-Caldés A, Cibeira MT, Bladé J, Prat A, Rosiñol L, Fernández de Larrea C. Nectin-2 Expression on Malignant Plasma Cells Is Associated with Better Response to TIGIT Blockade in Multiple Myeloma. Clin Cancer Res. 2020; 26:4688–98. 10.1158/1078-0432.CCR-19-3673 32513837

[11] Chauvin JM, Zarour HM. TIGIT in cancer immunotherapy. J Immunother Cancer. 2020; 8:e000957. 10.1136/jitc-2020-000957 32900861 PMC7477968

[12] Stamm H, Klingler F, Grossjohann EM, Muschhammer J, Vettorazzi E, Heuser M, Mock U, Thol F, Vohwinkel G, Latuske E, Bokemeyer C, Kischel R, Dos Santos C, et al. Immune checkpoints PVR and PVRL2 are prognostic markers in AML and their blockade represents a new therapeutic option. Oncogene. 2018; 37:5269–80. 10.1038/s41388-018-0288-y 29855615 PMC6160395

[13] Anand P, Guillaumet-Adkins A, Dimitrova V, Yun H, Drier Y, Sotudeh N, Rogers A, Ouseph MM, Nair M, Potdar S, Isenhart R, Kloeber JA, Vijaykumar T, et al. Single-cell RNA-seq reveals developmental plasticity with coexisting oncogenic states and immune evasion programs in ETP-ALL. Blood. 2021; 137:2463–80. 10.1182/blood.2019004547 33227818 PMC8109012

[14] Hoffman SE, Dowrey TW, Villacorta Martin C, Bi K, Titchen B, Johri S, DelloStritto L, Patel M, Mackichan C, Inga S, Chen J, Grimaldi G, Napolitano S, et al. Intertumoral lineage diversity and immunosuppressive transcriptional programs in well-differentiated gastroenteropancreatic neuroendocrine tumors. Sci Adv. 2023; 9:eadd9668. 10.1126/sciadv.add9668 37756410 PMC10530100

[15] Dama P, Tang M, Fulton N, Kline J, Liu H. Gal9/Tim-3 expression level is higher in AML patients who fail chemotherapy. J Immunother Cancer. 2019; 7:175. 10.1186/s40425-019-0611-3 31291985 PMC6621946

[16] Xiong Z, Ampudia Mesias E, Pluhar GE, Rathe SK, Largaespada DA, Sham YY, Moertel CL, Olin MR. CD200 Checkpoint Reversal: A Novel Approach to Immunotherapy. Clin Cancer Res. 2020; 26:232–41. 10.1158/1078-0432.CCR-19-2234 31624103

[17] Cieniewicz B, Uyeda MJ, Chen PP, Sayitoglu EC, Liu JM, Andolfi G, Greenthal K, Bertaina A, Gregori S, Bacchetta R, Lacayo NJ, Cepika AM, Roncarolo MG. Engineered type 1 regulatory T cells designed for clinical use kill primary pediatric acute myeloid leukemia cells. Haematologica. 2021; 106:2588–97. 10.3324/haematol.2020.263129 33054128 PMC8485690

[18] Flies AS, Darby JM, Lennard PR, Murphy PR, Ong CEB, Pinfold TL, De Luca A, Lyons AB, Woods GM, Patchett AL. A novel system to map protein interactions reveals evolutionarily conserved immune evasion pathways on transmissible cancers. Sci Adv. 2020; 6:eaba5031. 10.1126/sciadv.aba5031 32937435 PMC7458443

[19] Rawat AK, Pal K, Singh R, Anand A, Gupta S, Kishore D, Singh S, Singh RK. The CD200-CD200R cross-talk helps Leishmania donovani to down regulate macrophage and CD4^+^CD44^+^ T cells effector functions in an NFκB independent manner. Int J Biol Macromol. 2020; 151:394–401. 10.1016/j.ijbiomac.2020.02.189 32084478

[20] Kassiteridi C, Cole JE, Griseri T, Falck-Hansen M, Goddard ME, Seneviratne AN, Green PA, Park I, Shami AG, Pattarabanjird T, Upadhye A, Taylor AM, Handa A, et al. CD200 Limits Monopoiesis and Monocyte Recruitment in Atherosclerosis. Circ Res. 2021; 129:280–95. 10.1161/CIRCRESAHA.119.316062 33975450 PMC8260471

[21] Kim TJ, Park G, Kim J, Lim SA, Kim J, Im K, Shin MH, Fu YX, Del Rio ML, Rodriguez-Barbosa JI, Yee C, Suh KS, Kim SJ, et al. CD160 serves as a negative regulator of NKT cells in acute hepatic injury. Nat Commun. 2019; 10:3258. 10.1038/s41467-019-10320-y 31332204 PMC6646315

[22] Demerlé C, Gorvel L, Mello M, Pastor S, Degos C, Zarubica A, Angelis F, Fiore F, Nunes JA, Malissen B, Greillier L, Guittard G, Luche H, et al. Anti-HVEM mAb therapy improves antitumoral immunity both in vitro and in vivo, in a novel transgenic mouse model expressing human HVEM and BTLA molecules challenged with HVEM expressing tumors. J Immunother Cancer. 2023; 11:e006348. 10.1136/jitc-2022-006348 37230538 PMC10231015

[23] Liu W, Chou TF, Garrett-Thomson SC, Seo GY, Fedorov E, Ramagopal UA, Bonanno JB, Wang Q, Kim K, Garforth SJ, Kakugawa K, Cheroutre H, Kronenberg M, Almo SC. HVEM structures and mutants reveal distinct functions of binding to LIGHT and BTLA/CD160. J Exp Med. 2021; 218:e20211112. 10.1084/jem.20211112 34709351 PMC8558838

[24] Ware CF, Croft M, Neil GA. Realigning the LIGHT signaling network to control dysregulated inflammation. J Exp Med. 2022; 219:e20220236. 10.1084/jem.20220236 35604387 PMC9130030

[25] Kennedy R, Klein U. A T Cell-B Cell Tumor-Suppressive Axis in the Germinal Center. Immunity. 2019; 51:204–6. 10.1016/j.immuni.2019.07.006 31433965

[26] Wu F, Watanabe N, Tzioni MM, Akarca A, Zhang C, Li Y, Chen Z, Cucco F, Carmell N, Noh JY, Ito K, Dobson R, Moody S, et al. Thyroid MALT lymphoma: self-harm to gain potential T-cell help. Leukemia. 2021; 35:3497–508. 10.1038/s41375-021-01289-z 34021249 PMC8632687

[27] Van Laethem F, Donaty L, Tchernonog E, Lacheretz-Szablewski V, Russello J, Buthiau D, Almeras M, Moreaux J, Bret C. LAIR1, an ITIM-Containing Receptor Involved in Immune Disorders and in Hematological Neoplasms. Int J Mol Sci. 2022; 23:16136. 10.3390/ijms232416136 36555775 PMC9788452

[28] Peng DH, Rodriguez BL, Diao L, Chen L, Wang J, Byers LA, Wei Y, Chapman HA, Yamauchi M, Behrens C, Raso G, Soto LMS, Cuentes ERP, et al. Collagen promotes anti-PD-1/PD-L1 resistance in cancer through LAIR1-dependent CD8^+^ T cell exhaustion. Nat Commun. 2020; 11:4520. 10.1038/s41467-020-18298-8 32908154 PMC7481212

[29] Keerthivasan S, Şenbabaoğlu Y, Martinez-Martin N, Husain B, Verschueren E, Wong A, Yang YA, Sun Y, Pham V, Hinkle T, Oei Y, Madireddi S, Corpuz R, et al. Homeostatic functions of monocytes and interstitial lung macrophages are regulated via collagen domain-binding receptor LAIR1. Immunity. 2021; 54:1511–26.e8. 10.1016/j.immuni.2021.06.012 34260887

[30] Horn LA, Chariou PL, Gameiro SR, Qin H, Iida M, Fousek K, Meyer TJ, Cam M, Flies D, Langermann S, Schlom J, Palena C. Remodeling the tumor microenvironment via blockade of LAIR-1 and TGF-β signaling enables PD-L1-mediated tumor eradication. J Clin Invest. 2022; 132:e155148. 10.1172/JCI155148 35230974 PMC9012291

[31] Yang T, Qian Y, Liang X, Wu J, Zou M, Deng M. LILRB4, an immune checkpoint on myeloid cells. Blood Sci. 2022; 4:49–56. 10.1097/BS9.0000000000000109 35957669 PMC9362873

[32] Sharma N, Atolagbe OT, Ge Z, Allison JP. LILRB4 suppresses immunity in solid tumors and is a potential target for immunotherapy. J Exp Med. 2021; 218:e20201811. 10.1084/jem.20201811 33974041 PMC8117208

[33] Li Z, Deng M, Huang F, Jin C, Sun S, Chen H, Liu X, He L, Sadek AH, Zhang CC. LILRB4 ITIMs mediate the T cell suppression and infiltration of acute myeloid leukemia cells. Cell Mol Immunol. 2020; 17:272–82. 10.1038/s41423-019-0321-2 31700117 PMC7052276

[34] Yi M, Niu M, Xu L, Luo S, Wu K. Regulation of PD-L1 expression in the tumor microenvironment. J Hematol Oncol. 2021; 14:10. 10.1186/s13045-020-01027-5. 33413496 PMC7792099

[35] Agarwal S, Aznar MA, Rech AJ, Good CR, Kuramitsu S, Da T, Gohil M, Chen L, Hong SA, Ravikumar P, Rennels AK, Salas-Mckee J, Kong W, et al. Deletion of the inhibitory co-receptor CTLA-4 enhances and invigorates chimeric antigen receptor T cells. Immunity. 2023; 56:2388–407.e9. 10.1016/j.immuni.2023.09.001 37776850 PMC10591801

[36] Lorenzetti R, Janowska I, Smulski CR, Frede N, Henneberger N, Walter L, Schleyer MT, Hüppe JM, Staniek J, Salzer U, Venhoff A, Troilo A, Voll RE, et al. Abatacept modulates CD80 and CD86 expression and memory formation in human B-cells. J Autoimmun. 2019; 101:145–52. 10.1016/j.jaut.2019.04.016 31054942

[37] Ruffo E, Wu RC, Bruno TC, Workman CJ, Vignali DAA. Lymphocyte-activation gene 3 (LAG3): The next immune checkpoint receptor. Semin Immunol. 2019; 42:101305. 10.1016/j.smim.2019.101305 31604537 PMC6920665

[38] Heiduk M, Klimova A, Reiche C, Digomann D, Beer C, Aust DE, Distler M, Weitz J, Seifert AM, Seifert L. TIGIT Expression Delineates T-cell Populations with Distinct Functional and Prognostic Impact in Pancreatic Cancer. Clin Cancer Res. 2023; 29:2638–50. 10.1158/1078-0432.CCR-23-0258 37140899 PMC10345964

[39] Pan C, Zhai Y, Wang C, Liao Z, Wang D, Yu M, Wu F, Yin Y, Shi Z, Li G, Jiang T, Zhang W. Poliovirus receptor-based chimeric antigen receptor T cells combined with NK-92 cells exert potent activity against glioblastoma. J Natl Cancer Inst. 2024; 116:389–400. 10.1093/jnci/djad226 37944044 PMC10919341

[40] Murakami K, Miyatake S, Miyamae J, Saeki K, Shinya M, Akashi N, Mitsui I, Kobayashi K, Saeki K, Maeta N, Kanda T, Okamura Y, Hemmi H. Expression profile of immunoregulatory factors in canine tumors. Vet Immunol Immunopathol. 2022; 253:110505. 10.1016/j.vetimm.2022.110505 36327941

[41] Phong BL, Avery L, Sumpter TL, Gorman JV, Watkins SC, Colgan JD, Kane LP. Tim-3 enhances FcεRI-proximal signaling to modulate mast cell activation. J Exp Med. 2015; 212:2289–304. 10.1084/jem.20150388 26598760 PMC4689164

[42] Monney L, Sabatos CA, Gaglia JL, Ryu A, Waldner H, Chernova T, Manning S, Greenfield EA, Coyle AJ, Sobel RA, Freeman GJ, Kuchroo VK. Th1-specific cell surface protein Tim-3 regulates macrophage activation and severity of an autoimmune disease. Nature. 2002; 415:536–41. 10.1038/415536a 11823861

[43] Li Z, Ju Z, Frieri M. The T-cell immunoglobulin and mucin domain (Tim) gene family in asthma, allergy, and autoimmunity. Allergy Asthma Proc. 2013; 34:e21–6. 10.2500/aap.2013.34.3646 23406933

[44] Yang R, Sun L, Li CF, Wang YH, Yao J, Li H, Yan M, Chang WC, Hsu JM, Cha JH, Hsu JL, Chou CW, Sun X, et al. Galectin-9 interacts with PD-1 and TIM-3 to regulate T cell death and is a target for cancer immunotherapy. Nat Commun. 2021; 12:832. 10.1038/s41467-021-21099-2 33547304 PMC7864927

[45] Blom LH, Martel BC, Larsen LF, Hansen CV, Christensen MP, Juel-Berg N, Litman T, Poulsen LK. The immunoglobulin superfamily member CD200R identifies cells involved in type 2 immune responses. Allergy. 2017; 72:1081–90. 10.1111/all.13129 28106273

[46] Kotwica-Mojzych K, Jodłowska-Jędrych B, Mojzych M. CD200:CD200R Interactions and Their Importance in Immunoregulation. Int J Mol Sci. 2021; 22:1602. 10.3390/ijms22041602 33562512 PMC7915401

[47] Oumeslakht L, Aziz AI, Bensussan A, Ben Mkaddem S. CD160 receptor in CLL: Current state and future avenues. Front Immunol. 2022; 13:1028013. 10.3389/fimmu.2022.1028013 36420268 PMC9676924

[48] Liu S, Zhang W, Liu K, Wang Y. CD160 expression on CD8^+^ T cells is associated with active effector responses but limited activation potential in pancreatic cancer. Cancer Immunol Immunother. 2020; 69:789–97. 10.1007/s00262-020-02500-3 32055919 PMC11027829

[49] Sibilano R, Gaudenzio N, DeGorter MK, Reber LL, Hernandez JD, Starkl PM, Zurek OW, Tsai M, Zahner S, Montgomery SB, Roers A, Kronenberg M, Yu M, Galli SJ. A TNFRSF14-FcɛRI-mast cell pathway contributes to development of multiple features of asthma pathology in mice. Nat Commun. 2016; 7:13696. 10.1038/ncomms13696 27982078 PMC5171877

[50] Ning Z, Liu K, Xiong H. Roles of BTLA in Immunity and Immune Disorders. Front Immunol. 2021; 12:654960. 10.3389/fimmu.2021.654960 33859648 PMC8043046

[51] Carvalheiro T, Garcia S, Pascoal Ramos MI, Giovannone B, Radstake TRD, Marut W, Meyaard L. Leukocyte Associated Immunoglobulin Like Receptor 1 Regulation and Function on Monocytes and Dendritic Cells During Inflammation. Front Immunol. 2020; 11:1793. 10.3389/fimmu.2020.01793 32973751 PMC7466540

[52] Abdallah F, Coindre S, Gardet M, Meurisse F, Naji A, Suganuma N, Abi-Rached L, Lambotte O, Favier B. Leukocyte Immunoglobulin-Like Receptors in Regulating the Immune Response in Infectious Diseases: A Window of Opportunity to Pathogen Persistence and a Sound Target in Therapeutics. Front Immunol. 2021; 12:717998. 10.3389/fimmu.2021.717998 34594332 PMC8478328

[53] Jin AL, Zhang CY, Zheng WJ, Xian JR, Yang WJ, Liu T, Chen W, Li T, Wang BL, Pan BS, Li Q, Cheng JW, Wang PX, et al. CD155/SRC complex promotes hepatocellular carcinoma progression via inhibiting the p38 MAPK signalling pathway and correlates with poor prognosis. Clin Transl Med. 2022; 12:e794. 10.1002/ctm2.794 35384345 PMC8982318

[54] Zhan M, Zhang Z, Zhao X, Zhang Y, Liu T, Lu L, Li XY. CD155 in tumor progression and targeted therapy. Cancer Lett. 2022; 545:215830. 10.1016/j.canlet.2022.215830 35870689

[55] Zhu X, Liang R, Lan T, Ding D, Huang S, Shao J, Zheng Z, Chen T, Huang Y, Liu J, Pathak JL, Wei H, Wei B. Tumor-associated macrophage-specific CD155 contributes to M2-phenotype transition, immunosuppression, and tumor progression in colorectal cancer. J Immunother Cancer. 2022; 10:e004219. 10.1136/jitc-2021-004219 36104099 PMC9476138

[56] Wang Y, Luo YL, Chen YF, Lu ZD, Wang Y, Czarna A, Shen S, Xu CF, Wang J. Dually regulating the proliferation and the immune microenvironment of melanoma via nanoparticle-delivered siRNA targeting onco-immunologic CD155. Biomater Sci. 2020; 8:6683–94. 10.1039/d0bm01420f 33089844

[57] Sun Y, Luo J, Chen Y, Cui J, Lei Y, Cui Y, Jiang N, Jiang W, Chen L, Chen Y, Kuang Y, Tang K, Ke Z. Combined evaluation of the expression status of CD155 and TIGIT plays an important role in the prognosis of LUAD (lung adenocarcinoma). Int Immunopharmacol. 2020; 80:106198. 10.1016/j.intimp.2020.106198 31954274

[58] Ho DW, Tsui YM, Chan LK, Sze KM, Zhang X, Cheu JW, Chiu YT, Lee JM, Chan AC, Cheung ET, Yau DT, Chia NH, Lo IL, et al. Single-cell RNA sequencing shows the immunosuppressive landscape and tumor heterogeneity of HBV-associated hepatocellular carcinoma. Nat Commun. 2021; 12:3684. 10.1038/s41467-021-24010-1 34140495 PMC8211687

[59] Xu K, Wang R, Xie H, Hu L, Wang C, Xu J, Zhu C, Liu Y, Gao F, Li X, Wang C, Huang J, Zhou W, et al. Single-cell RNA sequencing reveals cell heterogeneity and transcriptome profile of breast cancer lymph node metastasis. Oncogenesis. 2021; 10:66. 10.1038/s41389-021-00355-6 34611125 PMC8492772

[60] Papanicolau-Sengos A, Yang Y, Pabla S, Lenzo FL, Kato S, Kurzrock R, DePietro P, Nesline M, Conroy J, Glenn S, Chatta G, Morrison C. Identification of targets for prostate cancer immunotherapy. Prostate. 2019; 79:498–505. 10.1002/pros.23756 30614027

[61] Mansorunov D, Apanovich N, Kipkeeva F, Nikulin M, Malikhova O, Stilidi I, Karpukhin A. The Correlation of Ten Immune Checkpoint Gene Expressions and Their Association with Gastric Cancer Development. Int J Mol Sci. 2022; 23:13846. 10.3390/ijms232213846 36430322 PMC9695628

[62] Shrestha M, Wang DY, Ben-David Y, Zacksenhaus E. CDK4/6 inhibitors and the pRB-E2F1 axis suppress PVR and PD-L1 expression in triple-negative breast cancer. Oncogenesis. 2023; 12:29. 10.1038/s41389-023-00475-1 37230983 PMC10213015

[63] You X, Liu F, Binder M, Vedder A, Lasho T, Wen Z, Gao X, Flietner E, Rajagopalan A, Zhou Y, Finke C, Mangaonkar A, Liao R, et al. Asxl1 loss cooperates with oncogenic Nras in mice to reprogram the immune microenvironment and drive leukemic transformation. Blood. 2022; 139:1066–79. 10.1182/blood.2021012519 34699595 PMC8854684

[64] Speiser DE, Ho PC, Verdeil G. Regulatory circuits of T cell function in cancer. Nat Rev Immunol. 2016; 16:599–611. 10.1038/nri.2016.80 27526640

[65] Thommen DS, Schumacher TN. T Cell Dysfunction in Cancer. Cancer Cell. 2018; 33:547–62. 10.1016/j.ccell.2018.03.012 29634943 PMC7116508

[66] Liu XP, Jin X, Seyed Ahmadian S, Yang X, Tian SF, Cai YX, Chawla K, Snijders AM, Xia Y, van Diest PJ, Weiss WA, Mao JH, Li ZQ, et al. Clinical significance and molecular annotation of cellular morphometric subtypes in lower-grade gliomas discovered by machine learning. Neuro Oncol. 2023; 25:68–81. 10.1093/neuonc/noac154 35716369 PMC9825346

[67] Haddad AF, Young JS, Oh JY, Okada H, Aghi MK. The immunology of low-grade gliomas. Neurosurg Focus. 2022; 52:E2. 10.3171/2021.11.FOCUS21587 35104791 PMC9283531

[68] Ben Aïssa A. Immunotherapy in Melanoma: Highlights for the General Practitioner. Praxis (Bern 1994). 2023; 112:135–42. 10.1024/1661-8157/a003972 36855885

[69] Wilden SM, Lang BM, Mohr P, Grabbe S. Checkpoint-Inhibitoren in der Immuntherapie: Ein Meilenstein in der Behandlung des malignen Melanoms. J Dtsch Dermatol Ges. 2016; 14:685–97. 10.1111/ddg.13012_g 27373243

[70] Jager MJ, Shields CL, Cebulla CM, Abdel-Rahman MH, Grossniklaus HE, Stern MH, Carvajal RD, Belfort RN, Jia R, Shields JA, Damato BE. Uveal melanoma. Nat Rev Dis Primers. 2020; 6:24. 10.1038/s41572-020-0158-0 32273508

[71] Durante MA, Rodriguez DA, Kurtenbach S, Kuznetsov JN, Sanchez MI, Decatur CL, Snyder H, Feun LG, Livingstone AS, Harbour JW. Single-cell analysis reveals new evolutionary complexity in uveal melanoma. Nat Commun. 2020; 11:496. 10.1038/s41467-019-14256-1 31980621 PMC6981133

[72] Zheng X, Xu H, Yi X, Zhang T, Wei Q, Li H, Ai J. Tumor-antigens and immune landscapes identification for prostate adenocarcinoma mRNA vaccine. Mol Cancer. 2021; 20:160. 10.1186/s12943-021-01452-1 34872584 PMC8645679

[73] Sharma P, Hu-Lieskovan S, Wargo JA, Ribas A. Primary, Adaptive, and Acquired Resistance to Cancer Immunotherapy. Cell. 2017; 168:707–23. 10.1016/j.cell.2017.01.017 28187290 PMC5391692

[74] Beer TM, Kwon ED, Drake CG, Fizazi K, Logothetis C, Gravis G, Ganju V, Polikoff J, Saad F, Humanski P, Piulats JM, Gonzalez Mella P, Ng SS, et al. Randomized, Double-Blind, Phase III Trial of Ipilimumab Versus Placebo in Asymptomatic or Minimally Symptomatic Patients With Metastatic Chemotherapy-Naive Castration-Resistant Prostate Cancer. J Clin Oncol. 2017; 35:40–7. 10.1200/JCO.2016.69.1584 28034081

[75] Kwon ED, Drake CG, Scher HI, Fizazi K, Bossi A, van den Eertwegh AJ, Krainer M, Houede N, Santos R, Mahammedi H, Ng S, Maio M, Franke FA, et al, and CA184-043 Investigators. Ipilimumab versus placebo after radiotherapy in patients with metastatic castration-resistant prostate cancer that had progressed after docetaxel chemotherapy (CA184-043): a multicentre, randomised, double-blind, phase 3 trial. Lancet Oncol. 2014; 15:700–12. 10.1016/S1470-2045(14)70189-5 24831977 PMC4418935

[76] Petitprez F, Fossati N, Vano Y, Freschi M, Becht E, Lucianò R, Calderaro J, Guédet T, Lacroix L, Rancoita PMV, Montorsi F, Fridman WH, Sautès-Fridman C, et al. PD-L1 Expression and CD8^+^ T-cell Infiltrate are Associated with Clinical Progression in Patients with Node-positive Prostate Cancer. Eur Urol Focus. 2019; 5:192–6. 10.1016/j.euf.2017.05.013 28753812

[77] Grosso JF, Kelleher CC, Harris TJ, Maris CH, Hipkiss EL, De Marzo A, Anders R, Netto G, Getnet D, Bruno TC, Goldberg MV, Pardoll DM, Drake CG. LAG-3 regulates CD8+ T cell accumulation and effector function in murine self- and tumor-tolerance systems. J Clin Invest. 2007; 117:3383–92. 10.1172/JCI31184 17932562 PMC2000807

[78] Sfanos KS, Bruno TC, Maris CH, Xu L, Thoburn CJ, DeMarzo AM, Meeker AK, Isaacs WB, Drake CG. Phenotypic analysis of prostate-infiltrating lymphocytes reveals TH17 and Treg skewing. Clin Cancer Res. 2008; 14:3254–61. 10.1158/1078-0432.CCR-07-5164 18519750 PMC3082357

[79] Davidsson S, Andren O, Ohlson AL, Carlsson J, Andersson SO, Giunchi F, Rider JR, Fiorentino M. FOXP3^+^ regulatory T cells in normal prostate tissue, postatrophic hyperplasia, prostatic intraepithelial neoplasia, and tumor histological lesions in men with and without prostate cancer. Prostate. 2018; 78:40–7. 10.1002/pros.23442 29105795 PMC5725695

[80] Piao YR, Piao LZ, Zhu LH, Jin ZH, Dong XZ. Prognostic value of T cell immunoglobulin mucin-3 in prostate cancer. Asian Pac J Cancer Prev. 2013; 14:3897–901. 10.7314/apjcp.2013.14.6.3897 23886204

[81] Wu J, Lin G, Zhu Y, Zhang H, Shi G, Shen Y, Zhu Y, Dai B, Ye D. Low TIM3 expression indicates poor prognosis of metastatic prostate cancer and acts as an independent predictor of castration resistant status. Sci Rep. 2017; 7:8869. 10.1038/s41598-017-09484-8 28827755 PMC5567055

