# Supplementary material for: Pan-cancer analysis of immune checkpoint receptors and ligands in various cells in the tumor immune microenvironment
Source: Aging (Albany NY). 2024 Aug 7;16(15):11683–728. doi: 10.18632/aging.206053 (PMC11346784; doi:10.18632/aging.206053)
Supplement: Supplementary Figures [file aging-16-206053-s001.pdf]

SUPPLEMENTARY FIGURES

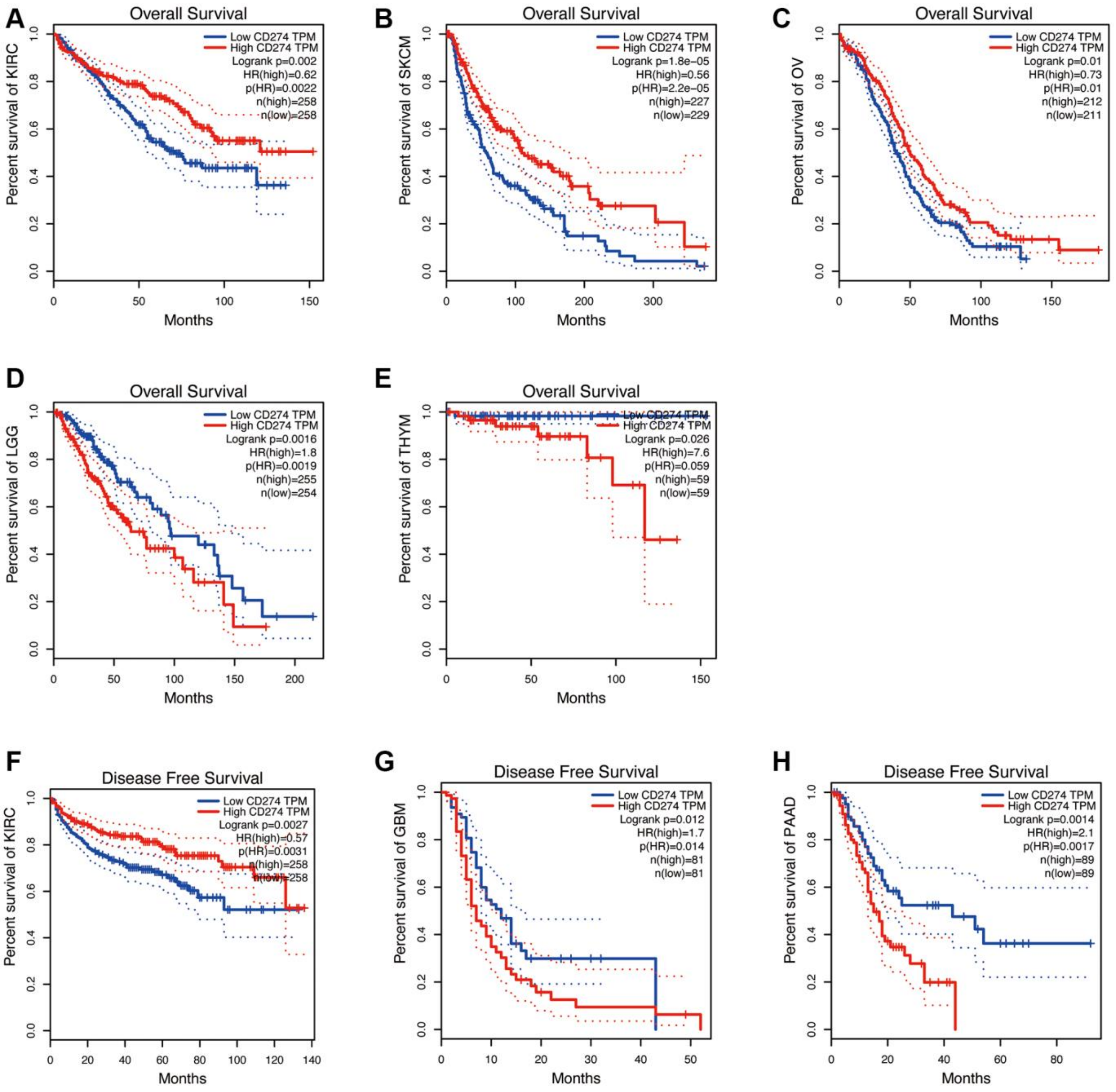

Supplementary Figure 1. A significant correlation between PDL1 expression and pan-cancer prognostic value. Expression of PDL1 was associated with overall survival (OS) in KIRC (**A**), SKCM (**B**), OV (**C**), LGG (**D**), THYM (**E**), and progression free survival (PFS) in KIRC (**F**), GBM (**G**), PAAD (**H**).

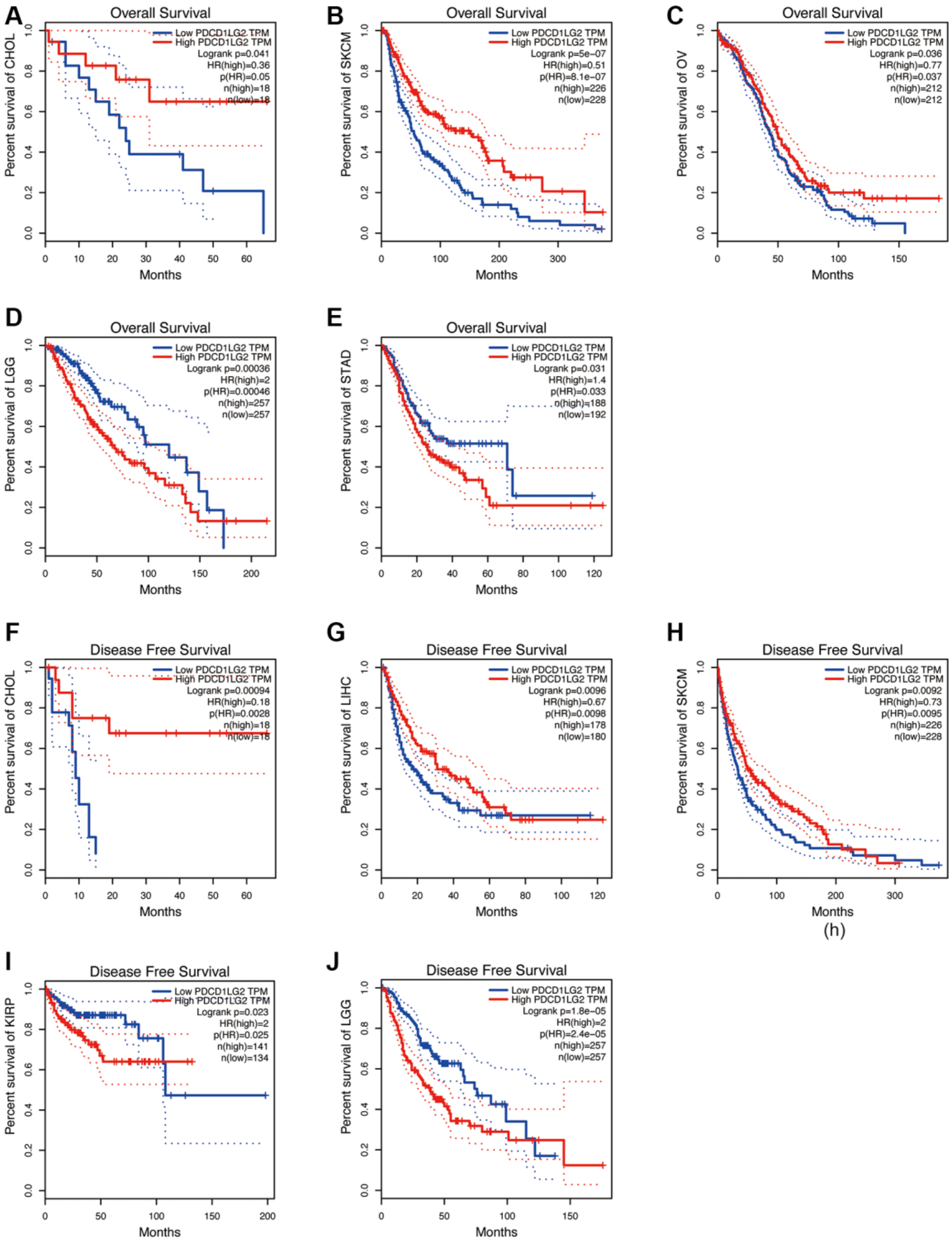

Supplementary Figure 2. A significant correlation between PDL2 expression and pan-cancer prognostic value. Expression of PDL2 was associated with overall survival (OS) in CHOL (**A**), SKCM (**B**), OV (**C**), LGG (**D**), STAD (**E**), and progression free survival (PFS) in CHOL (**F**), LIHC (**G**), SKCM (**H**), KIRP (**I**), LGG (**J**).

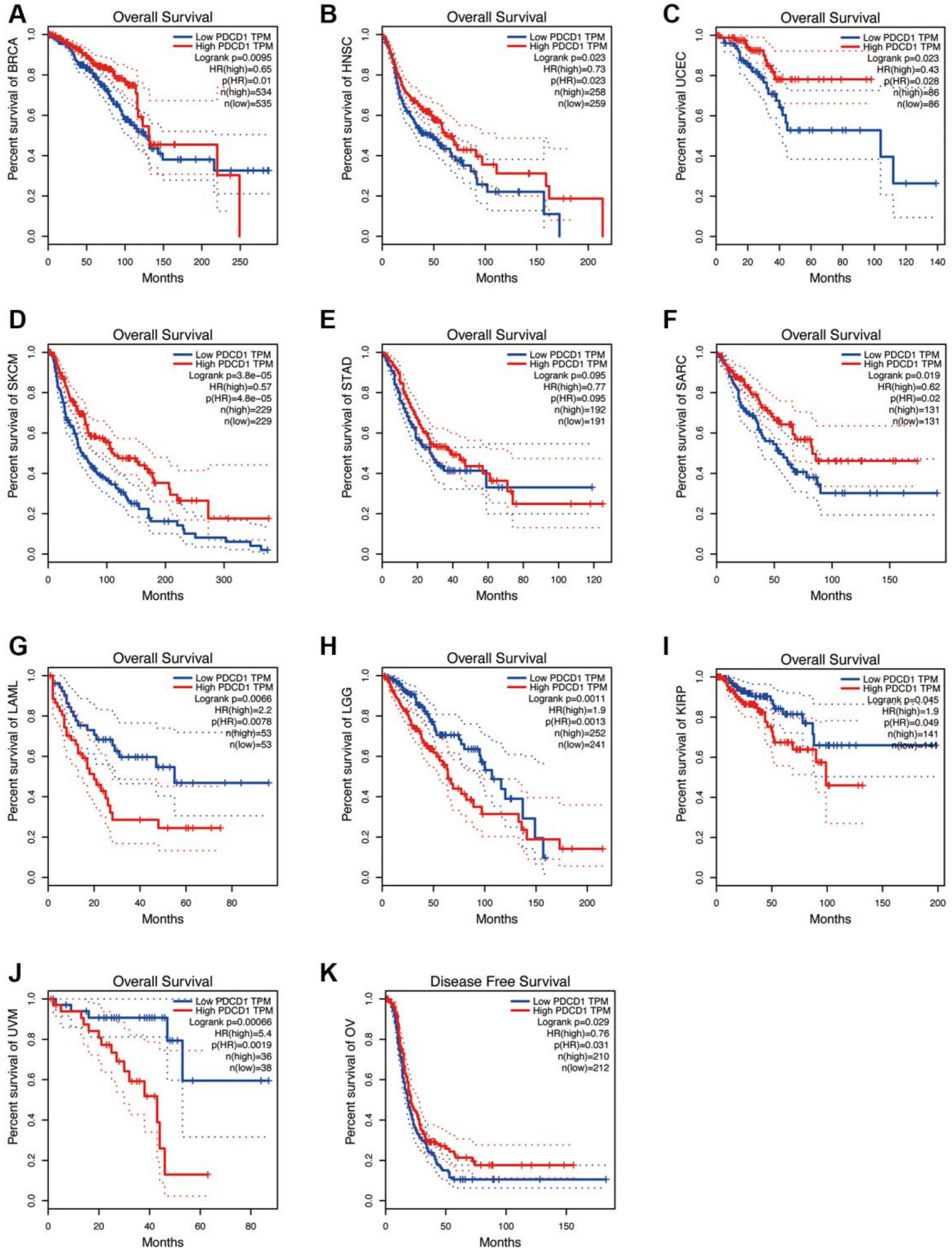

Supplementary Figure 3. A significant correlation between PD1 expression and pan-cancer prognostic value. Expression of PD1 was associated with overall survival (OS) in BRCA (**A**), HNSC (**B**), UCEC (**C**), SKCM (**D**), STAD (**E**), SARC (**F**), LAML (**G**), LGG (**H**), KIRP (**I**), UVM (**J**) and progression free survival (PFS) in OV (**K**).

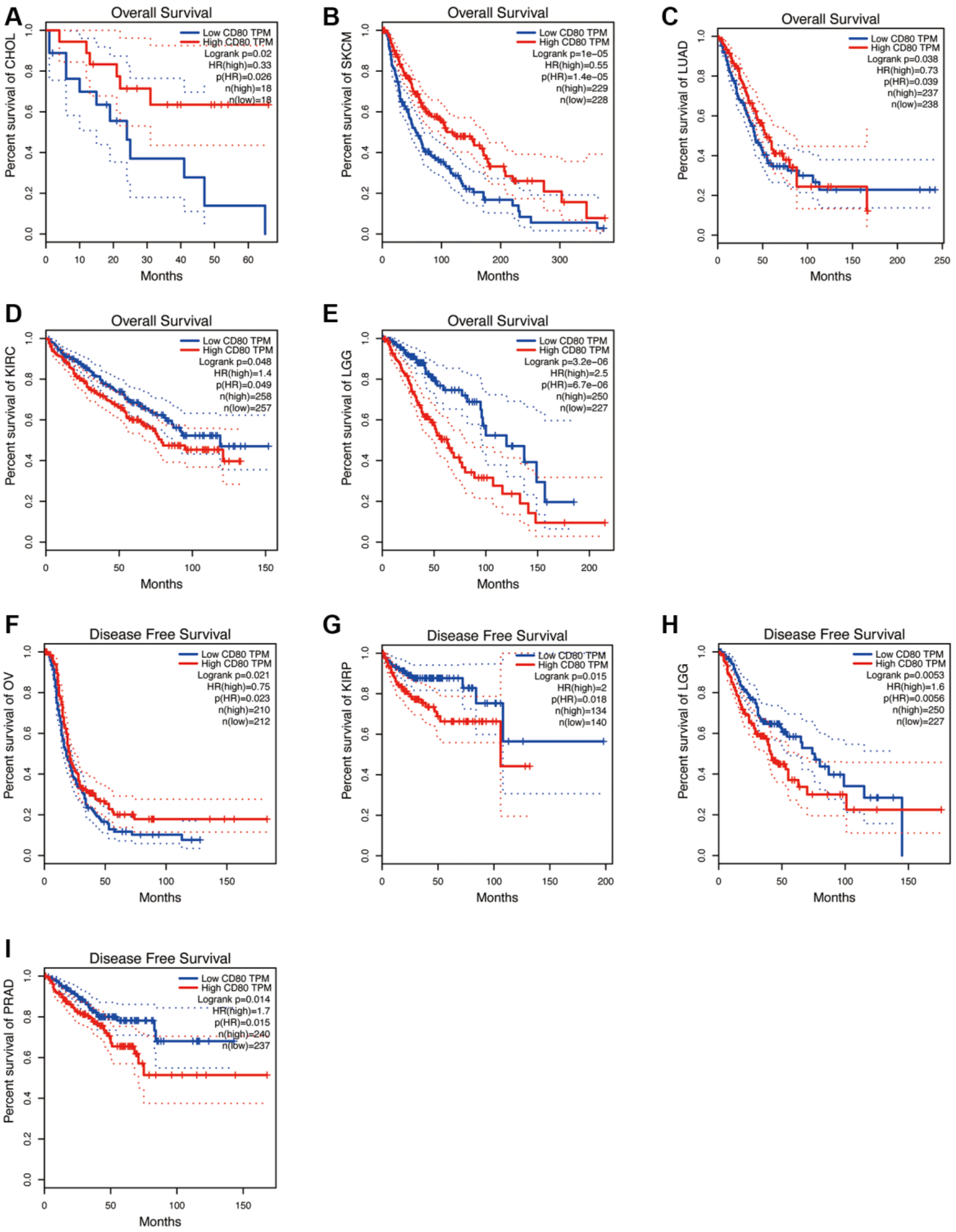

Supplementary Figure 4. A significant correlation between CD80 expression and pan-cancer prognostic value. Expression of CD80 was associated with overall survival (OS) in CHOL (**A**), SKCM (**B**), LUAD (**C**), KIRC (**D**), LGG (**E**), and progression free survival (PFS) in OV (**F**), KIRP (**G**), LGG (**H**), PRAD (**I**).

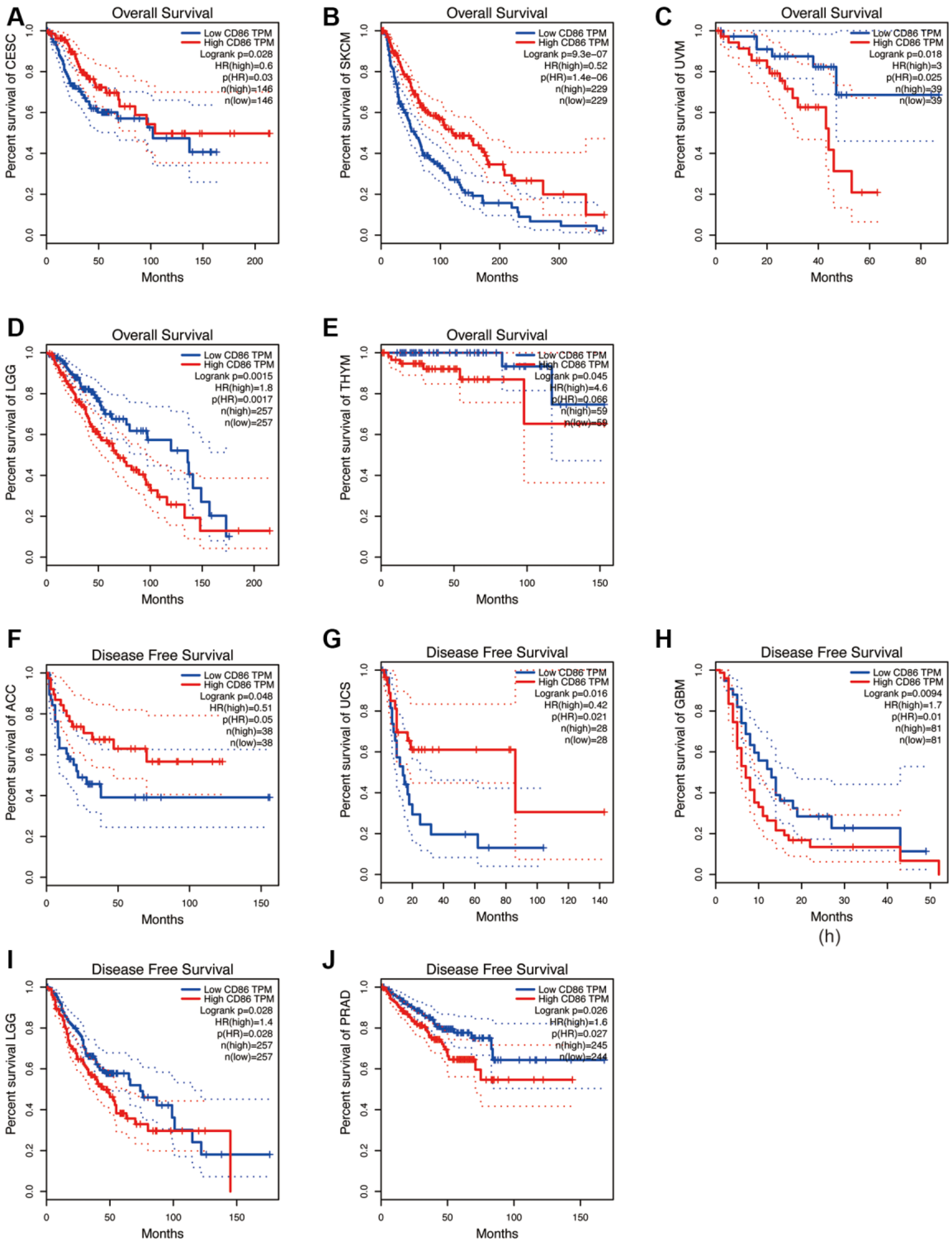

Supplementary Figure 5. A significant correlation between CD86 expression and pan-cancer prognostic value. Expression of CD86 was associated with overall survival (OS) in CESC (**A**), SKCM (**B**), UVM (**C**), LGG (**D**), THYM (**E**), and progression free survival (PFS) in ACC (**F**), UCS (**G**), GBM (**H**), LGG (**I**), PRAD (**J**).

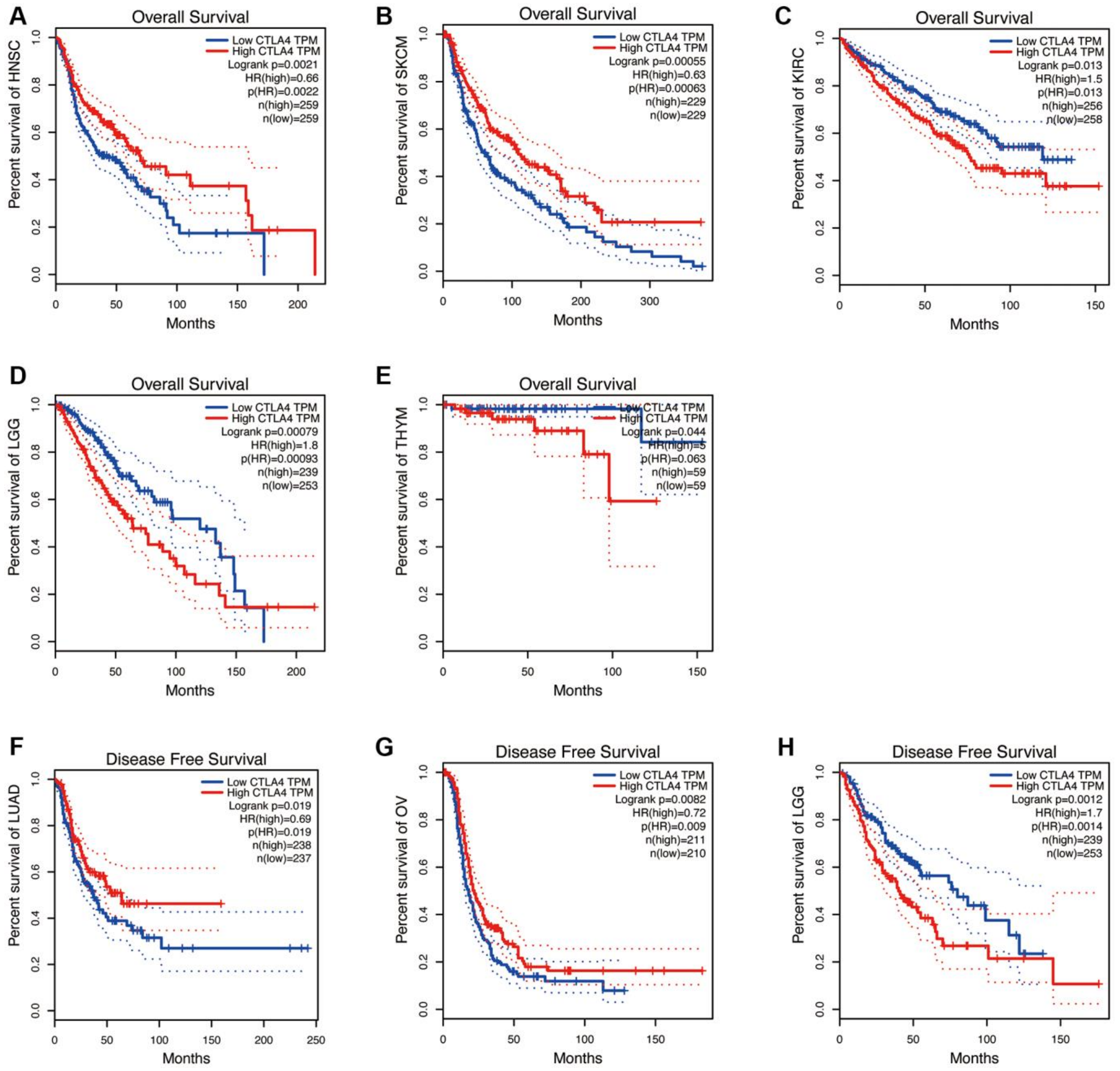

Supplementary Figure 6. A significant correlation between CTLA4 expression and pan-cancer prognostic value. Expression of CTLA4 was associated with overall survival (OS) in HNSC (**A**), SKCM (**B**), KIRC (**C**), LGG (**D**), THYM (**E**), and progression free survival (PFS) in LUAD (**F**), OV (**G**), LGG (**H**).

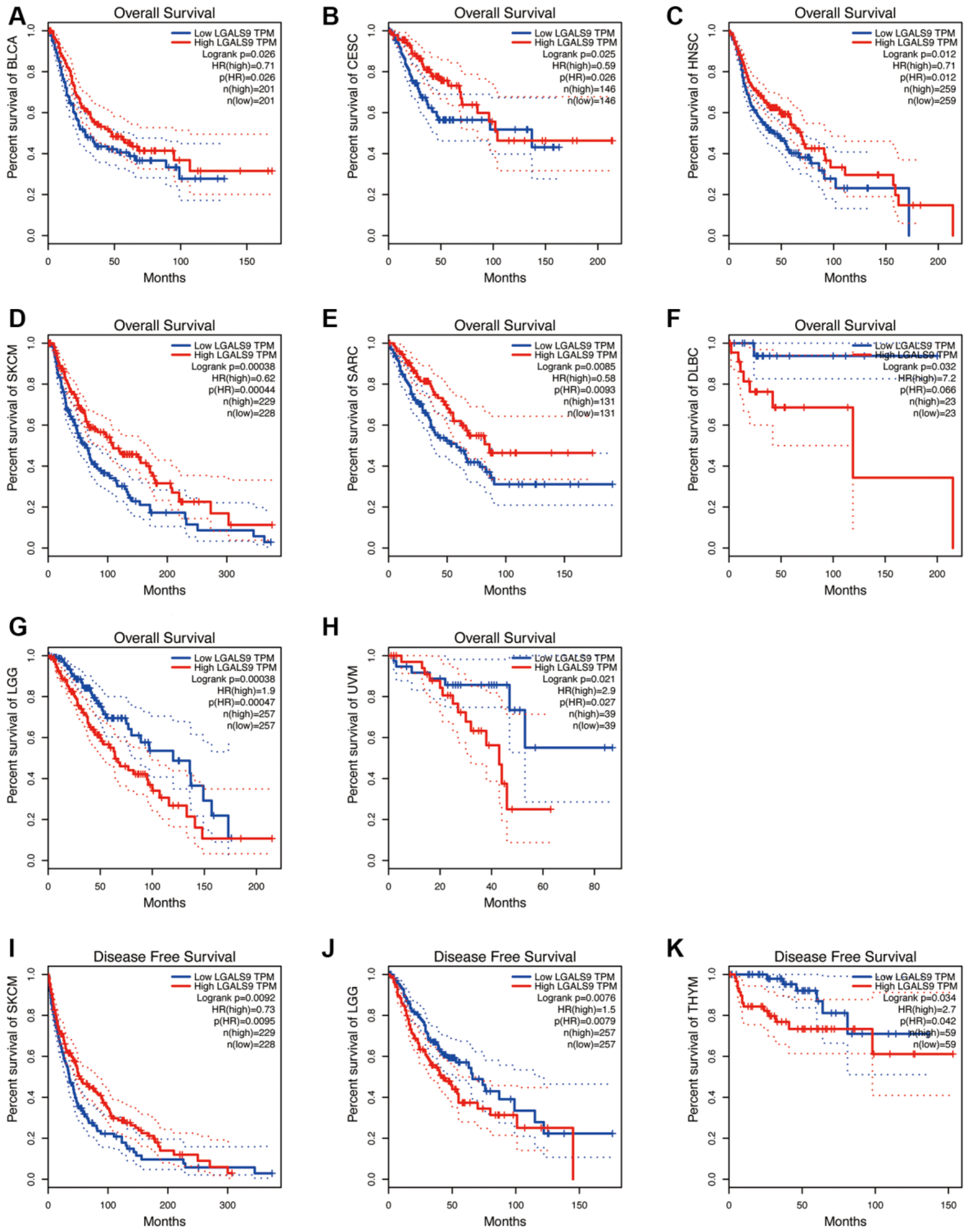

Supplementary Figure 7. A significant correlation between CD86 expression and pan-cancer prognostic value. Expression of CD86 was associated with overall survival (OS) in BLCA (**A**), CESC (**B**), HNSC (**C**), SKCM (**D**), SARC (**E**), DLBC (**F**) and progression free survival (PFS) in LGG (**G**), UVM (**H**), SKCM (**I**), LGG (**J**), THYM (**K**).

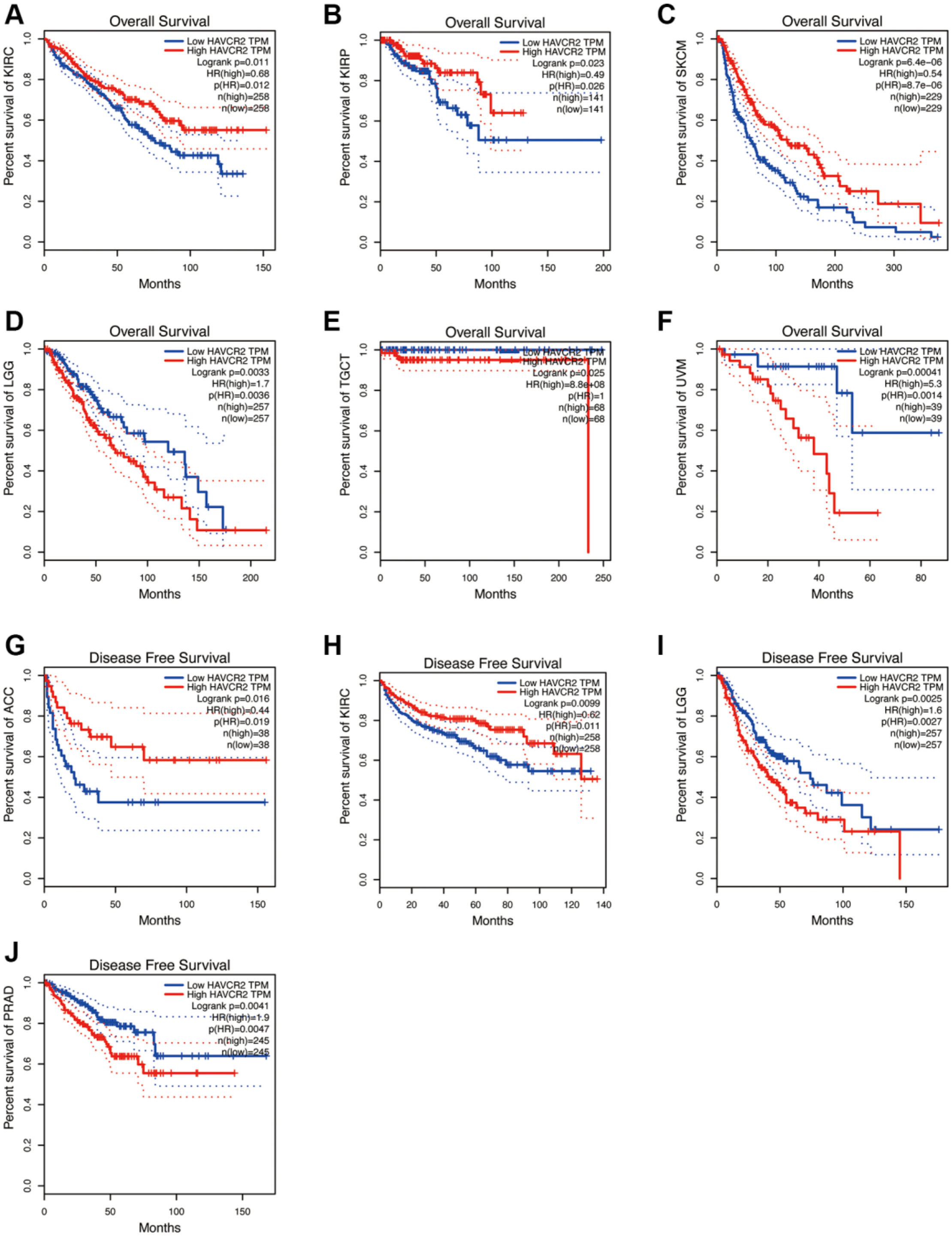

Supplementary Figure 8. A significant correlation between HAVCR2 expression and pan-cancer prognostic value. Expression of HAVCR2 was associated with overall survival (OS) in KIRC (**A**), KIRP (**B**), SKCM (**C**), LGG (**D**), TGCT (**E**), UVM (**F**) and progression free survival (PFS) in ACC (**G**), KIRC (**H**), LGG (**I**), PRAD (**J**).

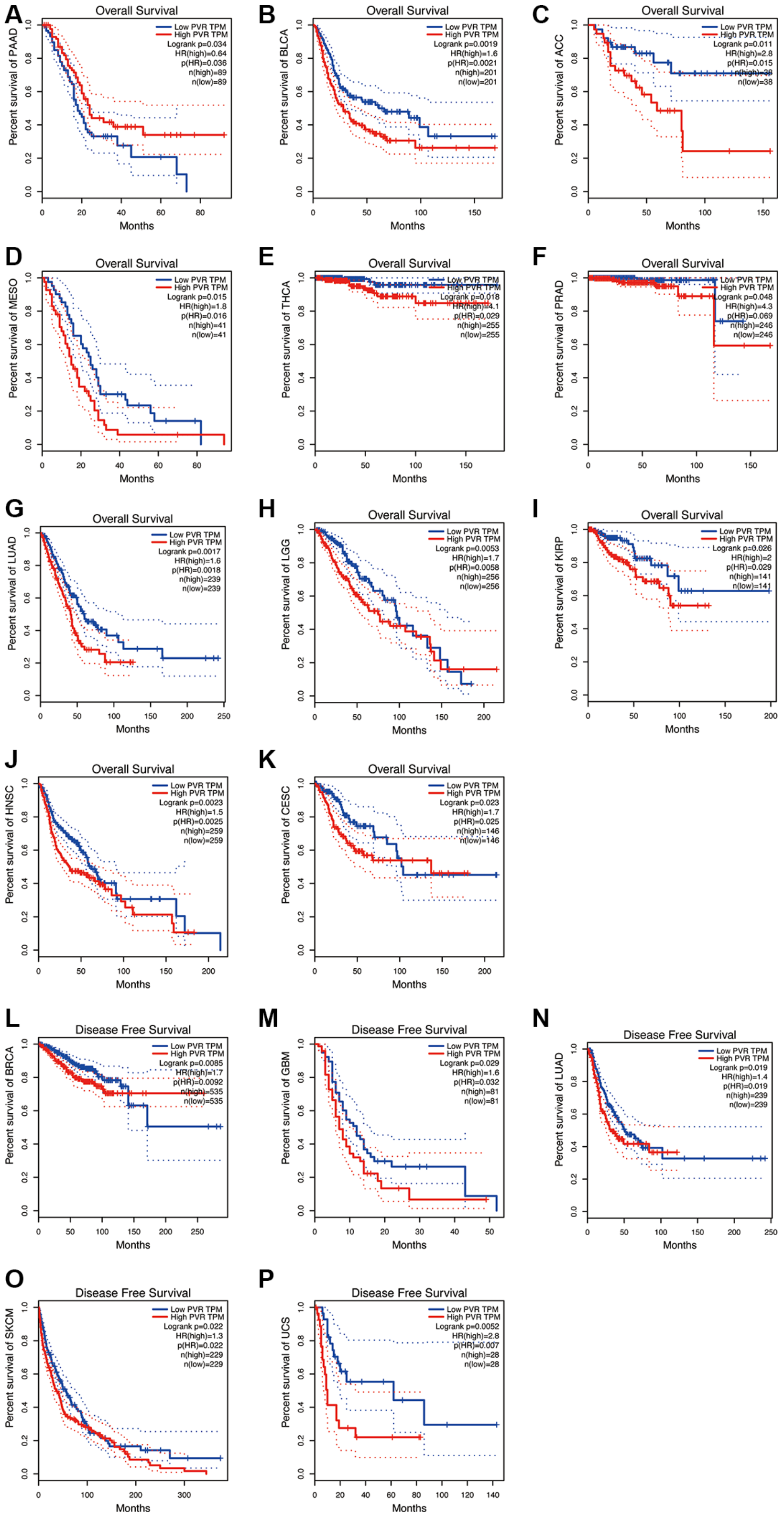

Supplementary Figure 9. A significant correlation between PVR expression and pan-cancer prognostic value. Expression of PVR was associated with overall survival (OS) in PAAD (**A**), BLCA (**B**), ACC (**C**), MESO (**D**), THCA (**E**), PRAD (**F**), LUAD (**G**), LGG (**H**), KIRP (**I**), HNSC (**J**), CESC (**K**) and progression free survival (PFS) in BRCA (**L**), GBM (**M**), LUAD (**N**), SKCM (**O**), UCS (**P**).

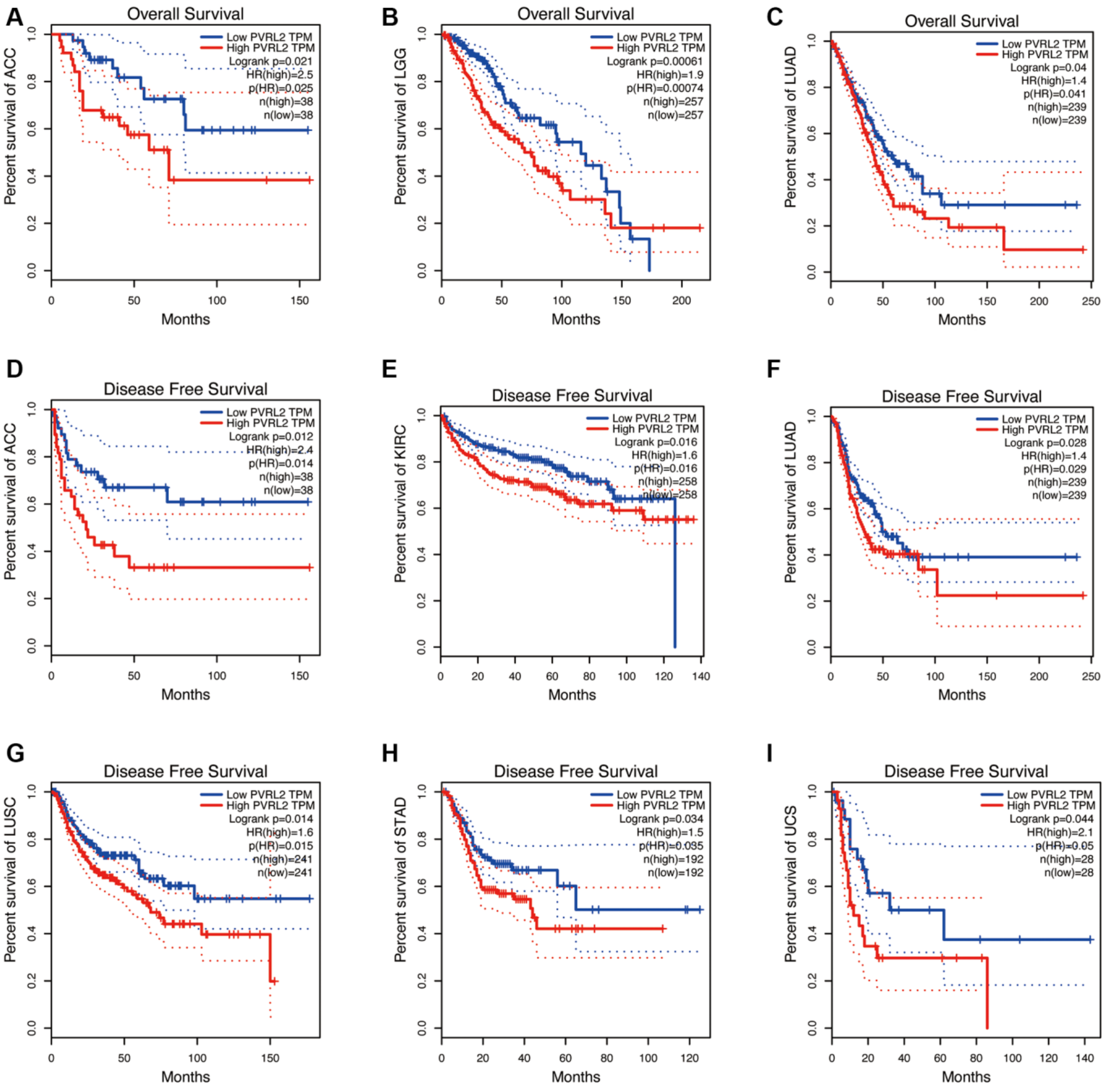

Supplementary Figure 10. A significant correlation between NECTIN2 expression and pan-cancer prognostic value. Expression of NECTIN2 was associated with overall survival (OS) in ACC (**A**), LGG (**B**), LUAD (**C**), and progression free survival (PFS) in ACC (**D**), KIRC (**E**), LUAD (**F**), LUSC (**G**), STAD (**H**), UCS (**I**).

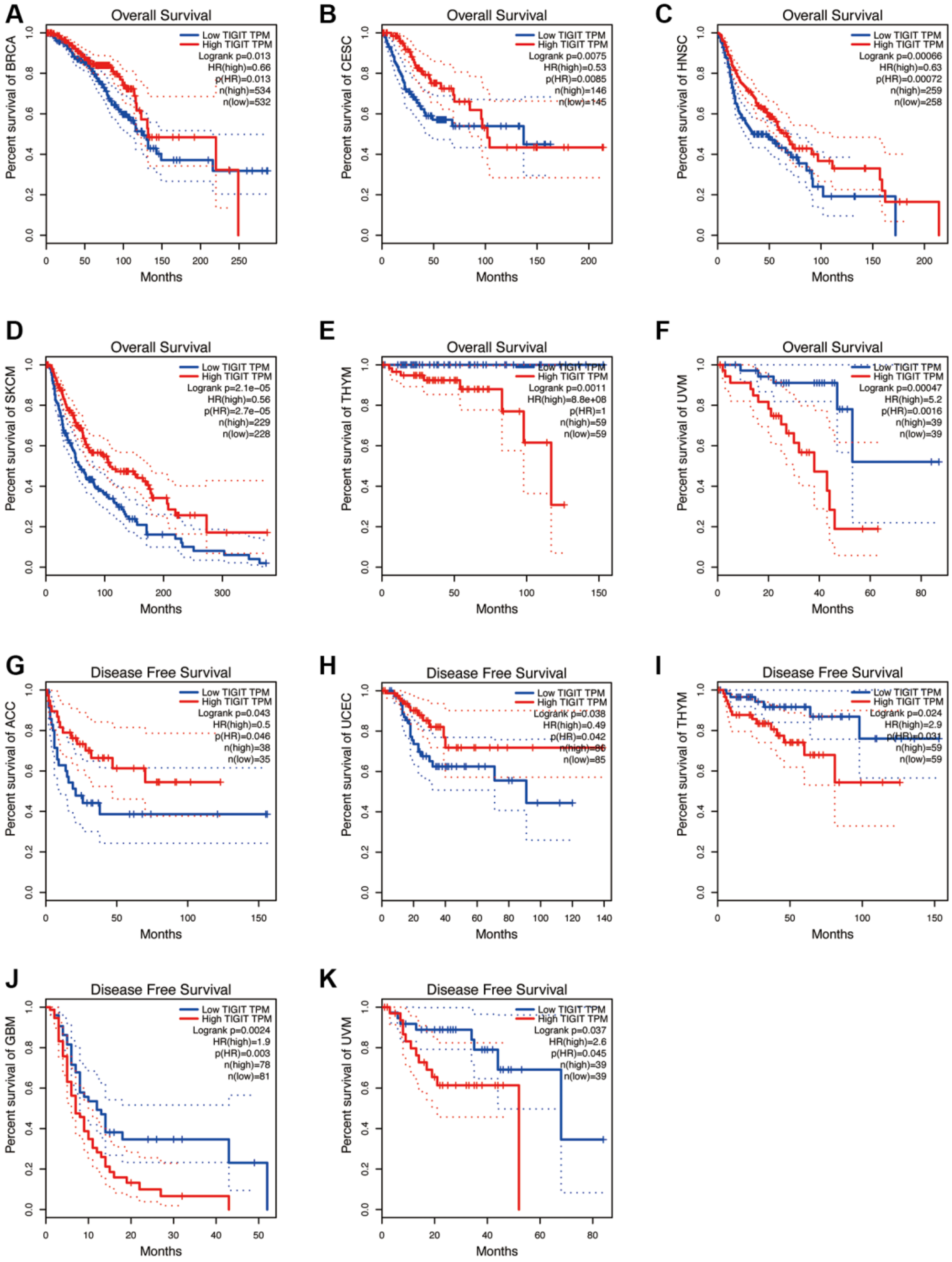

Supplementary Figure 11. A significant correlation between TIGIT expression and pan-cancer prognostic value. Expression of TIGIT was associated with overall survival (OS) in BRCA (**A**), CESC (**B**), HNSC (**C**), SKCM (**D**), THYM (**E**), UVM (**F**), and progression free survival (PFS) in ACC (**G**), UCEC (**H**), THYM (**I**), GBM (**J**), UVM (**K**).

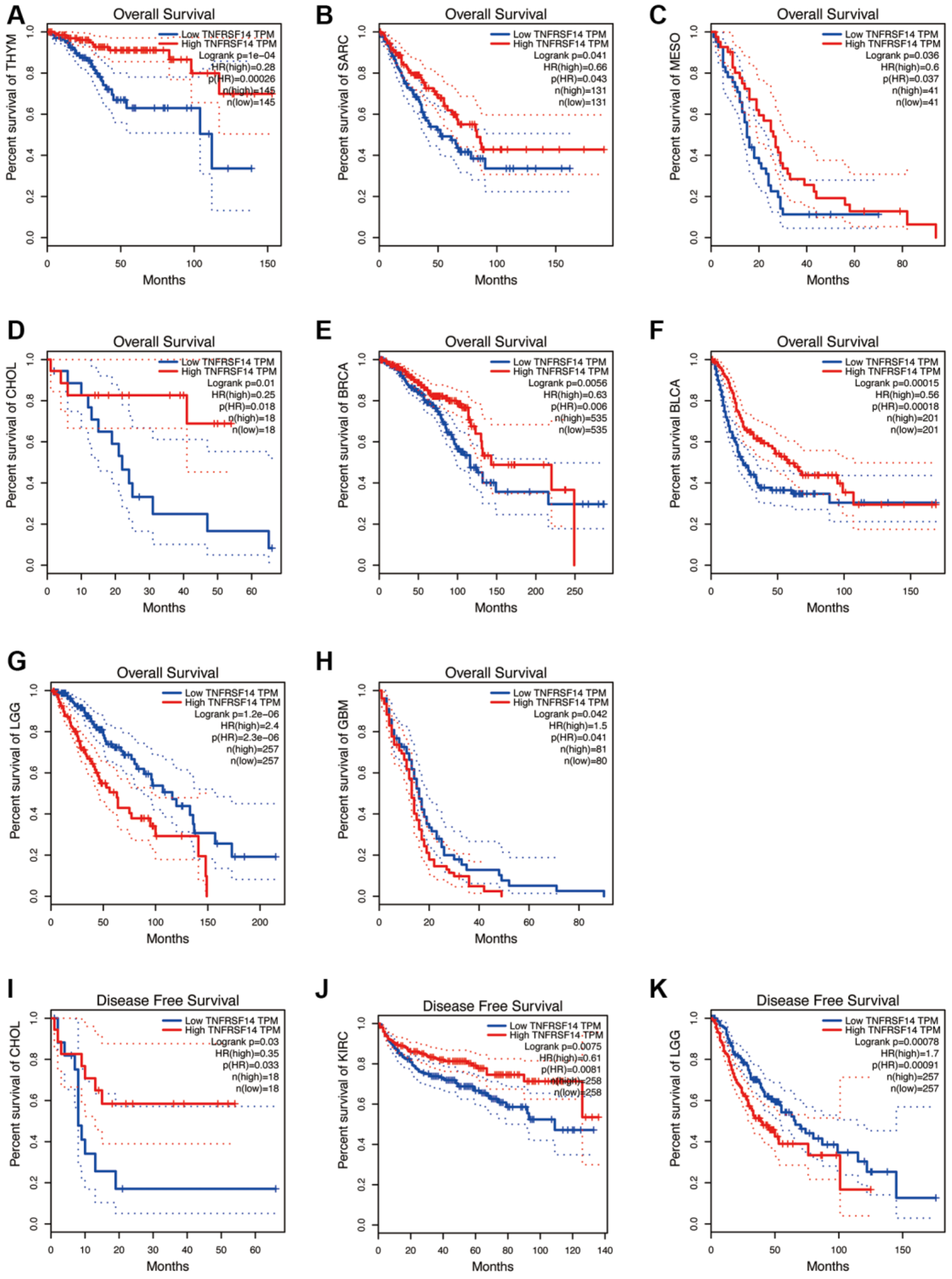

Supplementary Figure 12. A significant correlation between TNFRSF14 expression and pan-cancer prognostic value. Expression of TNFRSF14 was associated with overall survival (OS) in THYM (**A**), SARC (**B**), MESO (**C**), CHOL (**D**), BRCA (**E**), BLCA (**F**), LGG (**G**), GBM (**H**), and progression free survival (PFS) in CHOL (**I**), KIRC (**J**), LGG (**K**).

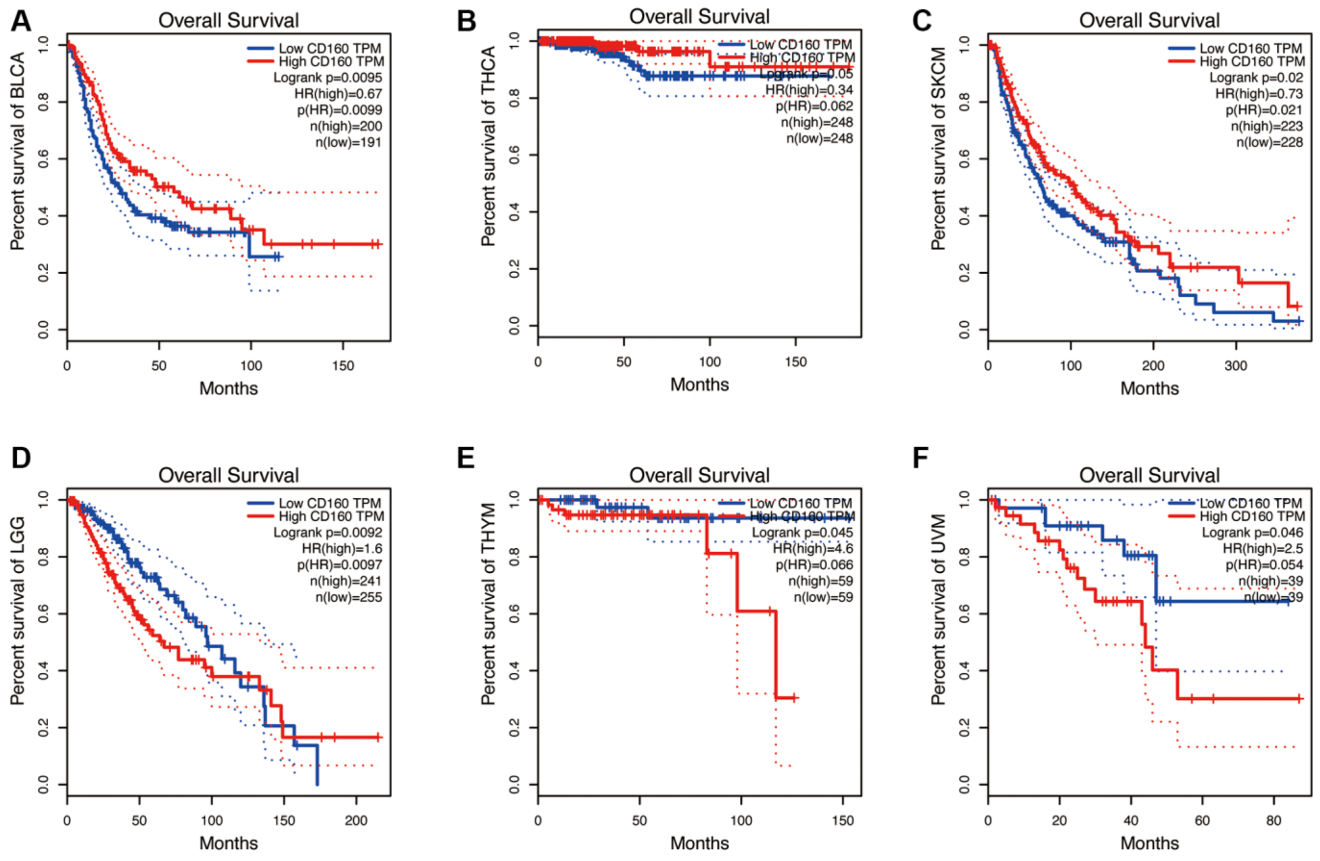

Supplementary Figure 13. A significant correlation between CD160 expression and pan-cancer prognostic value. Expression of CD160 was associated with overall survival (OS) in BLCA (**A**), THCA (**B**), SKCM (**C**), LGG (**D**), THYM (**E**), UVM (**F**).

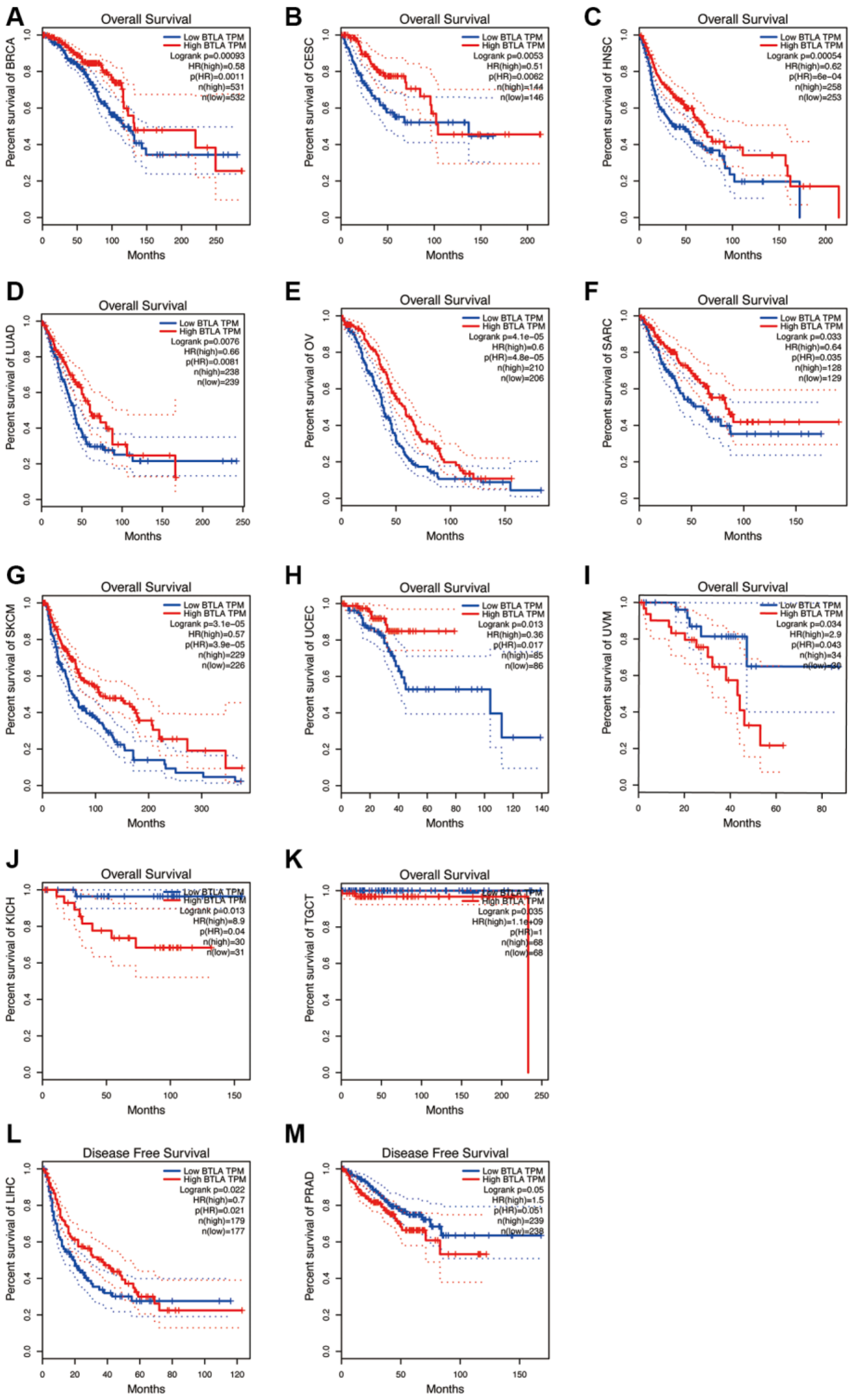

Supplementary Figure 14. A significant correlation between BTLA expression and pan-cancer prognostic value. Expression of BTLA was associated with overall survival (OS) in BRCA (**A**), CESC (**B**), HNSC (**C**), LUAD (**D**), OV (**E**), SARC (**F**), SKCM (**G**), UCEC (**H**), UVM (**I**), KICH (**J**), TGCT (**K**) and progression free survival (PFS) in LIHC (**L**), PRAD (**M**).

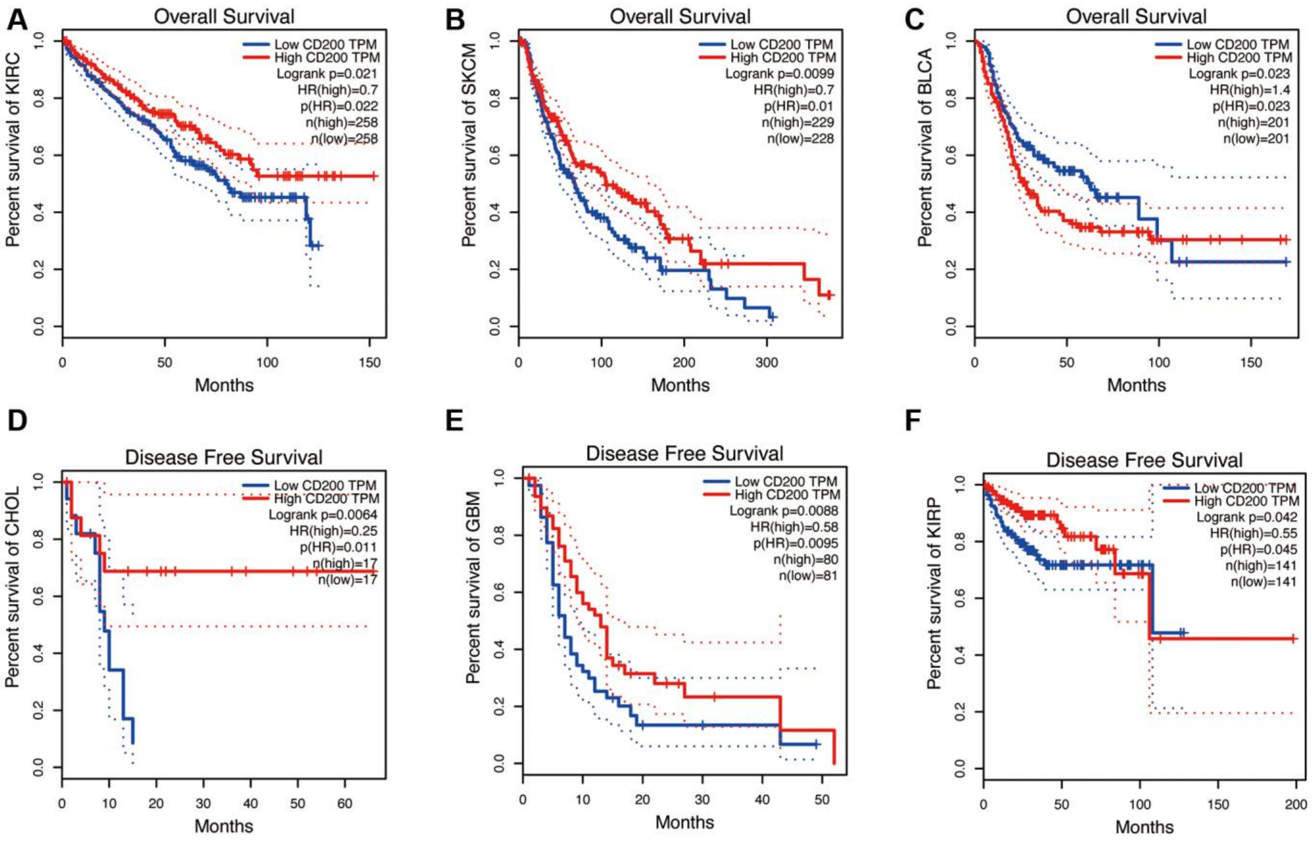

Supplementary Figure 15. A significant correlation between CD200 expression and pan-cancer prognostic value. Expression of CD200 was associated with overall survival (OS) in KIRC (**A**), SKCM (**B**), BLCA (**C**), and progression free survival (PFS) in CHOL (**D**), GBM (**E**), KIRP (**F**).

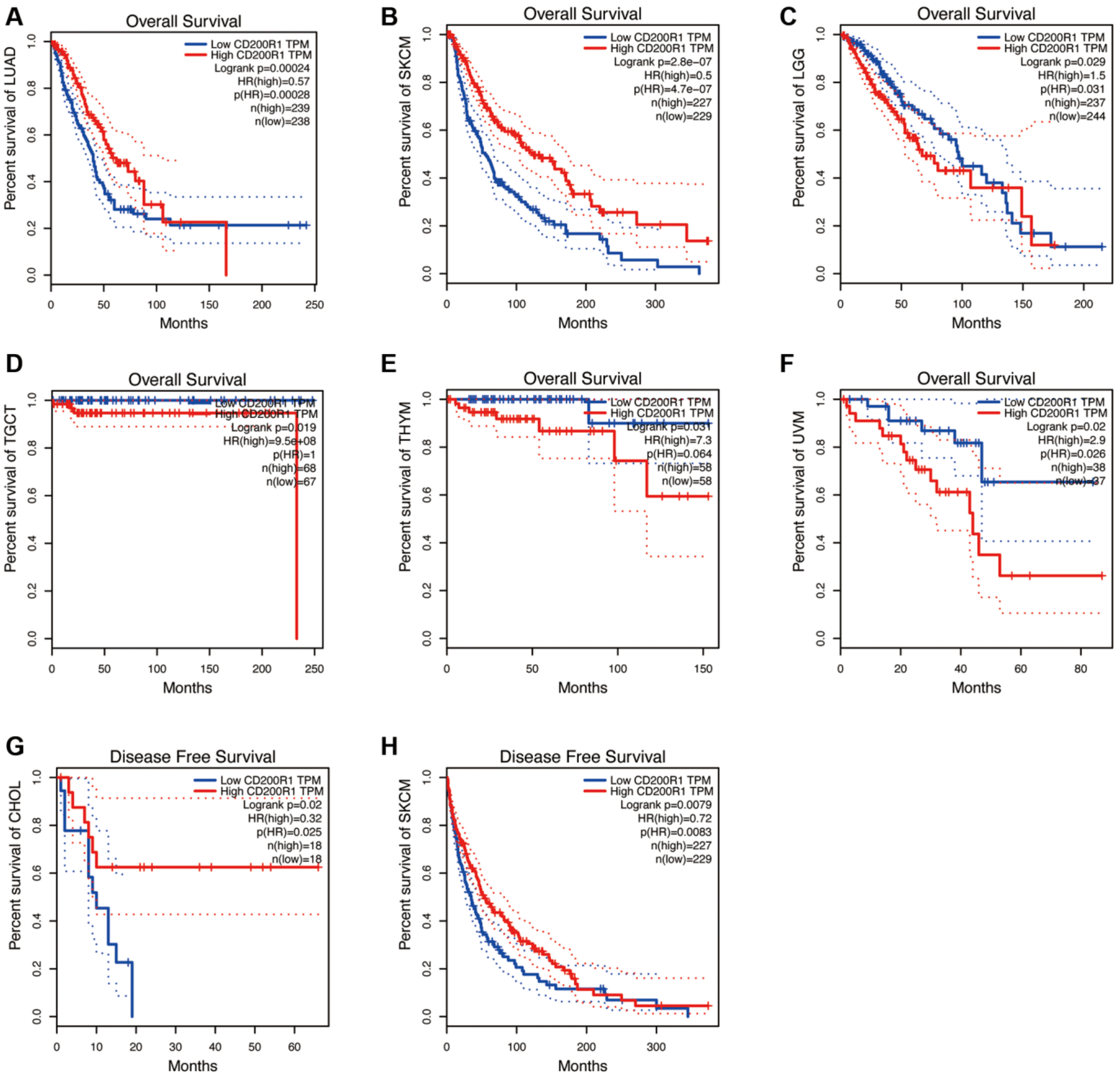

Supplementary Figure 16. A significant correlation between CD200R1 expression and pan-cancer prognostic value. Expression of CD200R1 was associated with overall survival (OS) in LUAD (**A**), SKCM (**B**), LGG (**C**), TGCT (**D**), THYM (**E**), UVM (**F**), and progression free survival (PFS) in CHOL (**G**), SKCM (**H**).

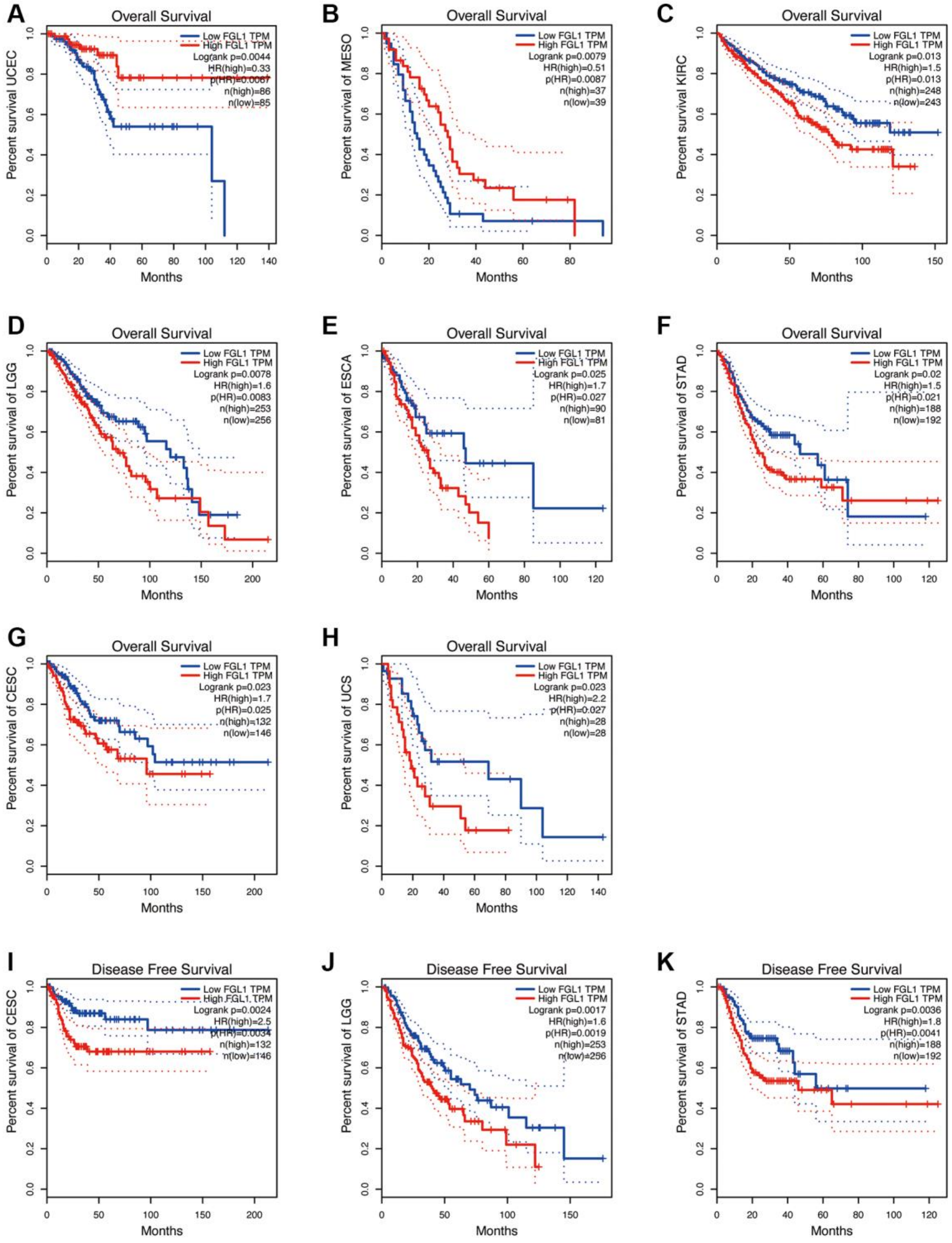

Supplementary Figure 17. A significant correlation between FGL1 expression and pan-cancer prognostic value. Expression of FGL1 was associated with overall survival (OS) in UCEC (**A**), MESO (**B**), KIRC (**C**), LGG (**D**), ESCA (**E**), STAD (**F**), CESC (**G**), UCS (**H**), and progression free survival (PFS) in CESC (**I**), LGG (**J**), STAD (**K**).

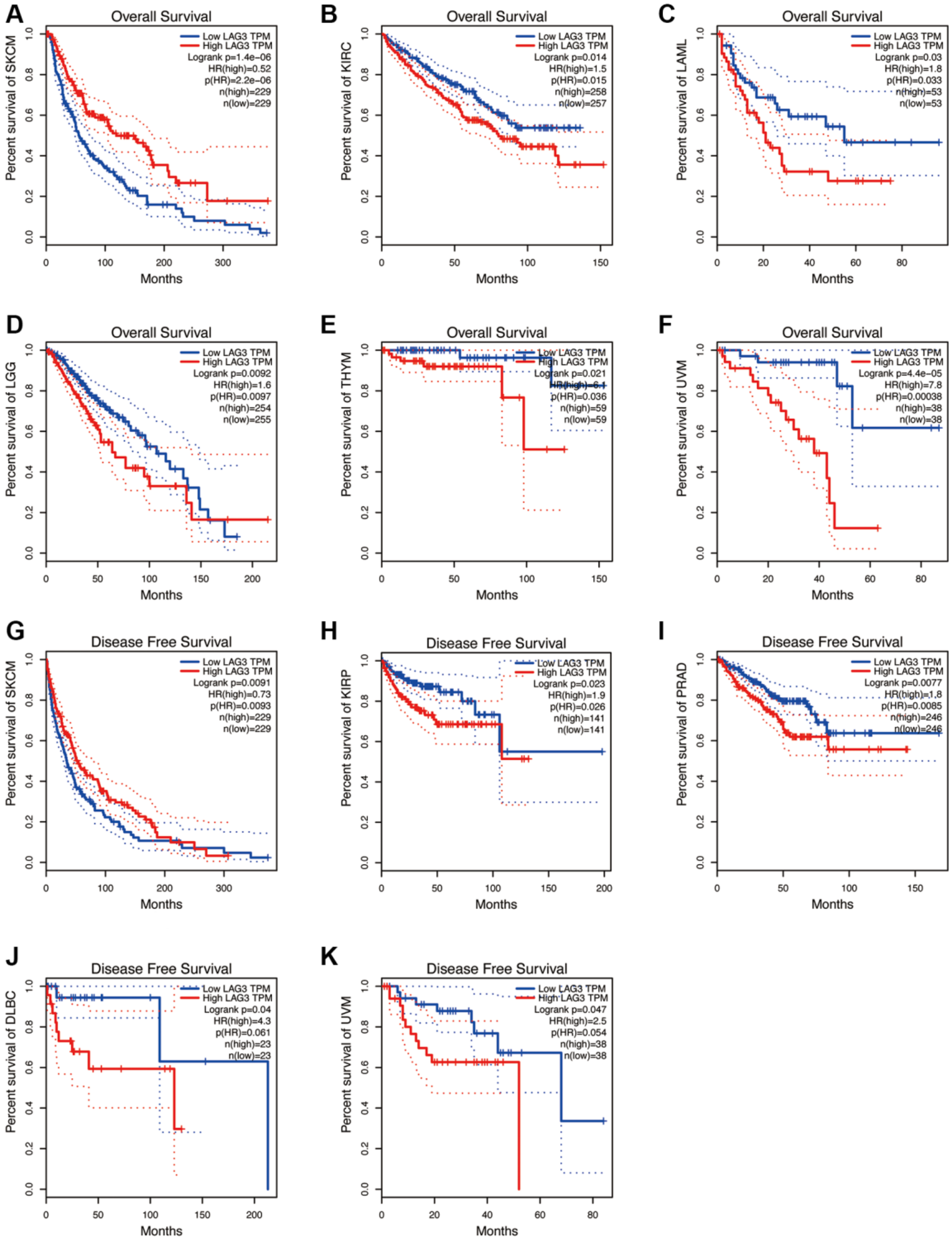

Supplementary Figure 18. A significant correlation between LAG3 expression and pan-cancer prognostic value. Expression of LAG3 was associated with overall survival (OS) in SKCM (**A**), KIRC (**B**), LAML (**C**), LGG (**D**), THYM (**E**), UVM (**F**), and progression free survival (PFS) in SKCM (**G**), KIRP (**H**), PRAD (**I**), DLBC (**J**), UVM (**K**).

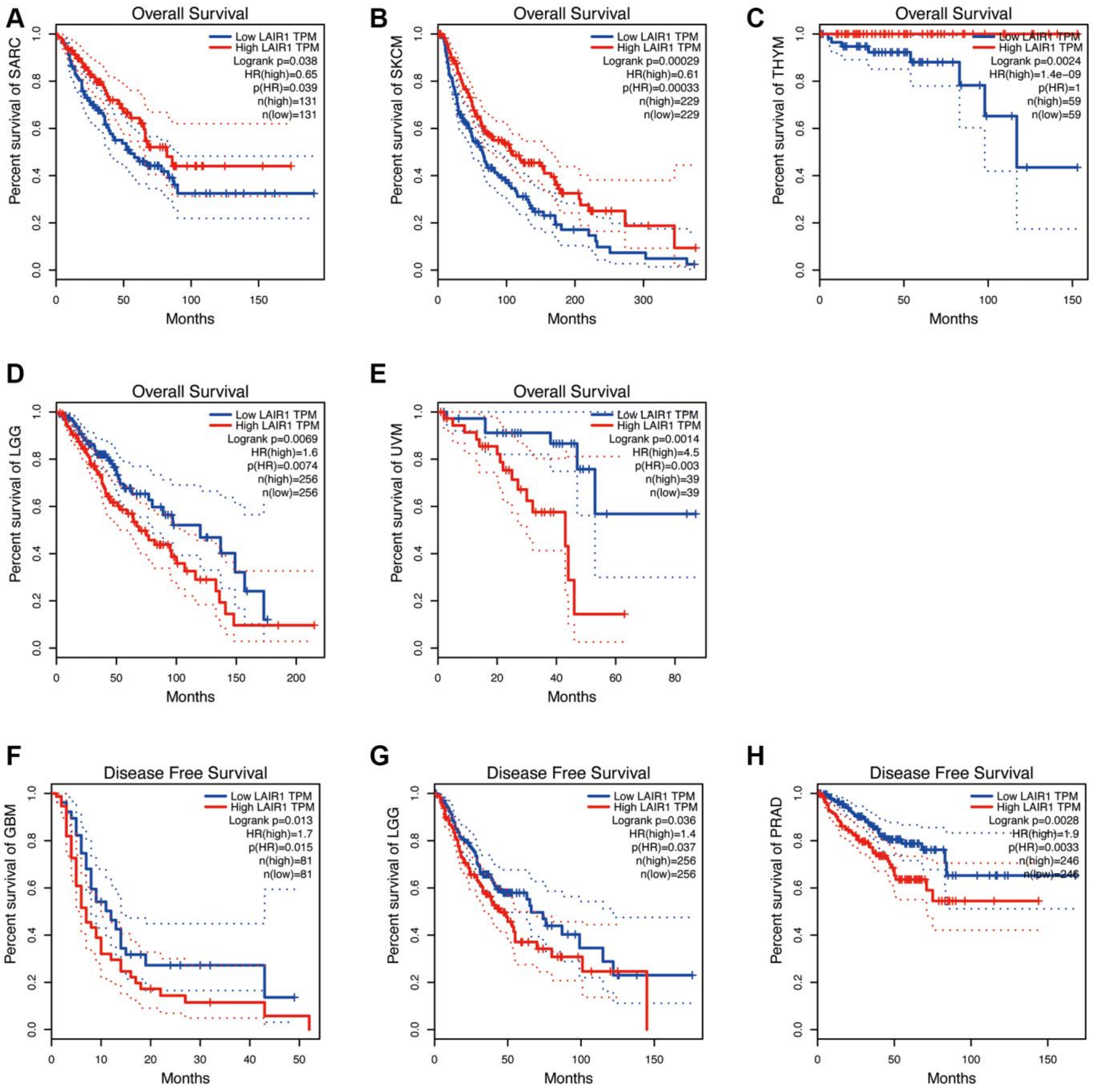

Supplementary Figure 19. A significant correlation between LAIR1 expression and pan-cancer prognostic value. Expression of LAIR1 was associated with overall survival (OS) in SARC (**A**), SKCM (**B**), THYM (**C**), LGG (**D**), UVM (**E**), and progression free survival (PFS) in GBM (**F**), LGG (**G**), PRAD (**H**).

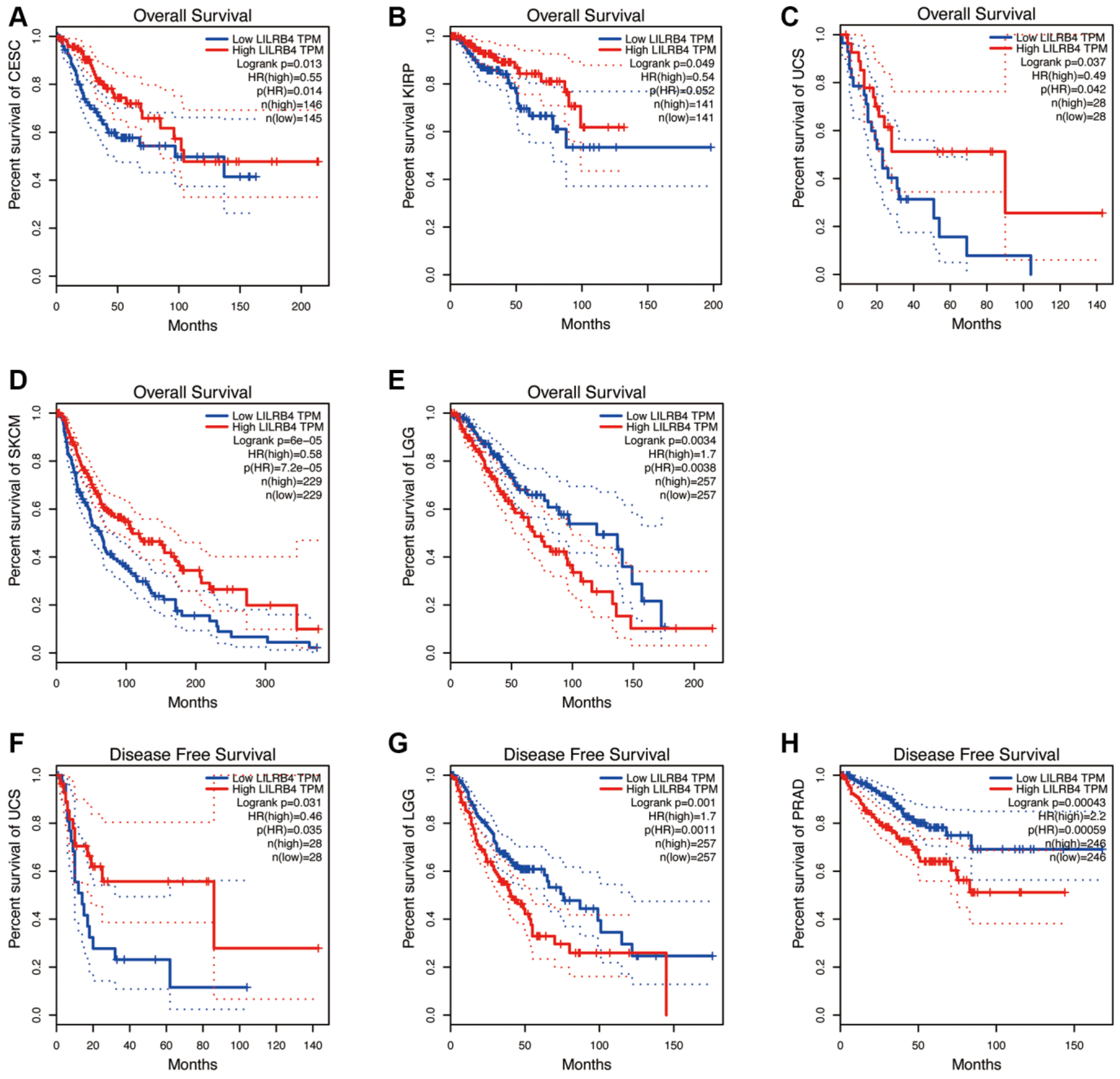

Supplementary Figure 20. A significant correlation between LILRB4 expression and pan-cancer prognostic value. Expression of LILRB4 was associated with overall survival (OS) in CESC (**A**), KIRP (**B**), UCS (**C**), SKCM (**D**), LGG (**E**), and progression free survival (PFS) in UCS (**F**), LGG (**G**), PRAD (**H**).
